# Oral lactoferrin reduces systemic inflammation, enhances anti-viral responses and modulates immune cell profiles: a randomised controlled trial in healthy, older adults

**DOI:** 10.1017/S000711452610631X

**Published:** 2026-05-14

**Authors:** Bronwyn S. Berthon, Evan J. Williams, Lily M. Williams, Kurtis F. Budden, Sarah A. Hiles, Nathan W. Bartlett, Lisa G. Wood

**Affiliations:** 1 School of Biomedical Sciences and Pharmacy, University of Newcastle, Callaghan, Australia; 2 Immune Health Research Program, Hunter Medical Research Institute, Kookaburra Circuit, New Lambton Heights, Australia; 3 School of Science, The University of Newcastle, Callaghan, Australia; 4 Infection Research Program, Hunter Medical Research Institute, New Lambton Heights, Australia

**Keywords:** Lactoferrin, immune function, systemic inflammation, randomised controlled trial

## Abstract

This randomised controlled trial examined the effect of a 4-week, high-dose (Lf-High, 600 mg/d) or low-dose (Lf-Low, 200 mg/d) oral lactoferrin (Lf) intervention *v*. placebo on immune cell responses to respiratory virus, immune cell subsets and systemic inflammation. In healthy older adults (*n* 103, ≥50 years old), *ex vivo* cytokine release of interferon (IFN)-α2, IFN-γ, IL-6 and TNF-α from rhinovirus A-16 (RV-16) or influenza A virus (H1N1) stimulated peripheral blood mononuclear cells, circulating immune cell subsets, and plasma IL-6, C-reactive protein (CRP) and TNF-α were assessed. Ninety-seven participants completed the 4-week intervention (Lf-High *n* 32, Lf-Low *n* 31, placebo *n* 34, withdrawals *n* 6). There was no difference in RV-16 or H1N1-induced IFN-γ release between groups. RV-16-induced IL-6 was lower in Lf-High *v*. placebo (*P* = 0·001), and RV-16-induced IFN-α2 was higher in Lf-High *v*. Lf-Low (*P* = 0·04). Lf-High increased total T cells (*P* = 0·03) and CD4^+^ T cells (*P* = 0·03) *v*. placebo. Lf-Low reduced neutrophil (*P* = 0·04), natural killer cell (*P* = 0·045), activated CD8^+^ T cell (*P* = 0·03) and γδ T cell (*P* = 0·03) frequency *v*. placebo. Plasma IL-6 (*P* = 0·004) and CRP (*P* = 0·03) were lower following Lf-High *v*. Lf-Low, but not placebo. Both high- and low-dose Lf altered *ex vivo* immune cell responses after 4 weeks. High-dose increased T-cell subsets, promoting adaptive immunity, and reduced systemic inflammation, while low-dose reduced proinflammatory and cytotoxic immune cells. High- and low-dose Lf supplements may have immunoceutical benefits in older adults.

In Australia^(1)^, and globally^(2)^, the incidence of acute respiratory infections is high, and since the beginning of the COVID-19 pandemic, interest in ‘immunoceuticals’, natural products which demonstrate immunomodulatory properties to enhance host immunity^(3)^, has burgeoned. Lactoferrin (Lf) is an immunoceutical that has received significant attention as a potential anti-viral treatment for acute respiratory infections. Lf is an iron-binding glycoprotein of the innate immune system, which is present in breastmilk, tears, bronchial secretions, gastrointestinal fluids and plasma^(4)^. In humans, circulating Lf concentrations are normally low, though are increased during infection and inflammation subsequent to neutrophil recruitment and release of Lf from activated neutrophil granules^(5)^. While endogenous Lf contributes to host defence through antiviral, antimicrobial, antioxidant and immunomodulatory activities^(6)^, bovine Lf, which is extracted from cow’s milk, can be used as an immunoceutical supplement and is generally regarded as safe and well tolerated^(7)^.

Lfs’ immuno-protective functions can be related to its iron-chelating abilities^(5)^, via direct and indirect effects on immune cells, or binding directly to receptors on virus and bacteria cell surfaces^(5,8,9)^. For example, human respiratory viruses such as coronaviruses utilise sugar-based molecular structures for cell binding: SARS-CoV-2 binds to heparan sulphate^(10)^. Evidence of anti-viral activity has been demonstrated through enhanced type I interferon (IFN) production, natural killer (NK) cell activity and type 1 T helper (T_H_1) cell cytokine responses^(11)^. Oral Lf has also been shown to have anti-inflammatory properties and reduce systemic inflammation^(12–14)^.

A small number of human trials have tested the immunomodulatory effects of Lf supplementation in various populations and health states, demonstrating reduced systemic inflammation, and reduced frequency/duration of respiratory virus symptoms^(15,16)^, with some studies showing improved immune function^(17,18)^ (*recently interrogated in a systematic review*)^(19)^. However, clinical studies examining the effect of Lf supplementation on immune function remain limited, with no consensus on appropriate dosing strategies. The variation in outcomes from clinical studies may be influenced by the timing and frequency of administration, the degree of iron saturation, reduced bioavailability due to enzymatic hydrolysis and various encapsulation delivery systems. In the absence of exposing participants to live virus infection or conducting lengthy and expensive surveillance trials, the effects of Lf on host immune response can be indirectly assessed *ex vivo* following supplementation, using primary human peripheral blood mononuclear cell (PBMC) culture models. PBMC isolated from whole blood can be cultured with virus *ex vivo*, and the immune cell response to stimulation can be determined^(20)^. Further, immune cell profiling can effectively examine the frequency and activation of circulating innate and adaptive immune cell populations, demonstrating direct immunomodulatory effects of Lf supplementation *in vivo*.

This study explored the immunomodulatory effects of Lf in healthy, older adults as immune function declines with increasing age, with a dampening of both adaptive and innate immune responses shown in older adults^(21)^. As Lf is proposed to improve immune function, older adults are a population who may be expected to benefit from the intervention. The study will serve as a dose-finding trial, investigating the effects of both high (600 mg/day) and low (200 mg/day) dose bovine Lf supplementation *v*. placebo on (i) immune cell responses to respiratory virus infection, (ii) peripheral immune cell subset frequency and (iii) systemic inflammation in healthy, older adults.

## Experimental methods

### Study design and population

The study was a double-blind, placebo-controlled, parallel-group, 4-week randomised (1:1:1) controlled, dose-finding trial, testing the effects of bovine Lf supplementation in healthy, older adults on immune cell responses to respiratory virus infection, immune cell subset frequency and systemic inflammation outcomes. The 4-week study duration was determined by previous oral Lf intervention trials showing changes in *ex vivo* immune cell responses^(22)^, peripheral immune cell subsets^(17,18)^ and systemic inflammation^(23–25)^ within a 4-week period. Thus, a 4-week intervention duration was considered appropriate to answer the aims of this trial. The trial was conducted at the Hunter Medical Research Institute Newcastle, Australia, from August 2022 to October 2023. Healthy^(26)^ older adults (≥50 years of age), with no acute or serious illness, were recruited through volunteer databases and by advertisement. Exclusion criteria included cow’s milk allergy, current smoking (within 6 months), use of products containing Lf, daily use (or use within previous 4 weeks of randomisation) of systemic corticosteroid, immunosuppressive, antibiotic or antiviral drugs, unstable cardiac, renal, pulmonary, endocrine, immunological or neurological disorders, acute or terminal illness, HIV or active cancer and any vaccination within previous 4 weeks before randomisation. Irregular use (other than daily administration) of dietary or nutritional supplements was also excluded. Baseline visits for participants using supplements inconsistently, or those containing Lf, were postponed for at least 2–4 weeks to provide a washout period before commencing the trial (water-soluble vitamins and herbs – 2 weeks; fat-soluble vitamins, fish oil, probiotics, prebiotics and synbiotics – 4 weeks). To maintain external validity to community-dwelling older adults, non-communicable diseases or concomitant medications were not excluded in this trial^(27)^. Baseline visits were postponed for at least 4 weeks for any participant who required antibiotic, antiviral or immune-modulating drugs (oral corticosteroids) within the previous month, if they were currently experiencing an acute illness, or following vaccination (including seasonal influenza vaccines and COVID-19 vaccines). In addition, participants were asked to avoid scheduling vaccination appointments during the trial 4-week intervention period. Participants were withdrawn from the study if: indicated by the occurrence and nature of an adverse event, or the participant or the participant’s general practitioner requested that the participant be withdrawn, or the participant refused/was unable to comply with the requirements of the protocol, or if eligibility status changed during the study following the diagnosis of a new condition or due to treatment with excluded treatments/medications. The full trial protocol and the Statistical Analysis Plan can be accessed in the Supplementary Material. Consumers were not involved in the planning, design or conduct of the study.

### Ethical approval

The trial was conducted in accordance with the guidelines laid down in the Declaration of Helsinki, ICH Code of Good Clinical Practice standards and the National Health and Medical Research Council National Statement of Ethical Conduct in Research. All procedures involving human participants were approved by the Hunter New England Human Research Ethics Committee (2021/ETH10928). Written informed consent was obtained from all participants. The trial was registered with the Australian and New Zealand Clinical Trials Registry (ACTRN12621001511820, https://www.anzctr.org.au, 05/11/2021) and the Therapeutic Goods Administration (CT-2021-CTN-04015-1-v1).

### Intervention

Participants were randomised to either the high-dose (Lf-High, 600 mg/day) Lf group (2 × 300 mg capsules), the low-dose (Lf-Low, 200 mg/day) Lf group (2 × 100 mg capsules) or placebo group (2 × placebo capsules). All participants were instructed to consume 2 capsules per day with water, in the morning, 30 min before food, for a 4-week period. Participants consumed the final intervention dose in the morning, on the day of the follow up assessment visit. The Lf intervention capsules contained bovine Lf powder with an Fe saturation of 16 % and Fe concentration of 12·1 mg/100 mg (PUREnFERRIN, Noumi Limited, Shepparton), extracted from Australian cow’s milk. The colour of Lf-Low capsules was identical to Lf-High capsules, and the weight of Lf-Low capsules was matched to Lf-High capsules with excipient (200 mg microcrystalline cellulose). Identical placebo capsules (285 mg microcrystalline cellulose, 15 mg *Beta vulgaris* root powder containing 5 % maltodextrin) were manufactured. The intervention capsules were manufactured according to GMP in a TGA approved facility by BJP Laboratories Pty Ltd (Yatala) and packaged by Complementary Medicines Group (Warriewood). The Lf and placebo interventions were encapsulated into size 00, white gelatine capsules and packaged in white, opaque 150 mL bottles with tamper seals.

Adherence to the intervention was monitored and assessed using the trial diary and pill countback of remaining capsules at the follow-up assessment visit. Participants were asked to complete the diary each day by recording capsule consumption. Participants who consumed at least 80 % of the intervention were adherent to the intervention, determined by pill count back.

### Procedures

Participants were screened via telephone and then attended the baseline assessment visit. Medical and vaccination history, smoking history, pack years, current medications and supplement use were recorded, and eligibility was confirmed before randomisation. Participants completed an online FFQ to assess usual dietary intake over the previous 12 months (DQES v3.2, Victorian Cancer Council)^(28)^. A study diary was provided to participants to record daily adherence to the intervention and adverse events. Two weeks after randomisation, participants were contacted by telephone for motivational purposes and to collect information on adherence, adverse events or illness. Four weeks (28 days) after the baseline visit, participants attended the clinic for follow-up assessment.

Clinical assessment at baseline and follow-up included body weight (Nuweigh EB8271; Newcastle Weighing Services), height using a wall-mounted stadiometer (Seca 220; Seca) and fasting (12-hour) venous blood collection via venepuncture. BMI was calculated as follows: body weight (kg)/height (m)^2^. Participants also completed the US Centers for Disease Control and Prevention Health-related quality of life questionnaire-14 at each assessment visit to monitor health status and well-being^(29,30)^.

### Outcomes

The primary outcome of the study was the difference in *ex vivo* interferon-γ (IFN-γ) release by virus-stimulated PBMC between baseline and follow-up. Secondary outcomes included the difference in peripheral immune cell subset frequency (eosinophils, neutrophils, T cells, CD (cluster of differentiation) 4^+^ T cells, CD8^+^ T cells, activated CD4^+^ T cells, activated CD8^+^ T cells and regulatory T cells (Tregs), γδ T cells, B cells, NK cells, blood dendritic cell antigen-1 (BDCA-1) dendritic cells (DC), BDCA-3 DC, plasmacytoid DC (pDC) and systemic inflammatory biomarkers (plasma IL-6, C-reactive protein (CRP), TNF-α). All outcomes were assessed at baseline and follow up.

### Laboratory techniques

#### Blood sample processing

Venous blood samples were collected in 9 mL EDTA anticoagulant collection tubes. Blood samples were centrifuged at ∼1700 × ***g*** for 10 min at 4°C. Plasma was collected and stored at −80°C for later analyses.

#### Ex vivo peripheral blood mononuclear cell isolation and culture

PBMC were isolated from the remaining cellular fraction via density gradient centrifugation using SepMate™ tubes (STEMCELL Technologies) and LymphoprepTM (STEMCELL Technologies) as per manufacturer’s specifications. PBMC were counted and assessed for viability by staining with trypan blue 0·4 %, then resuspended in Roswell Park Memorial Institute (RPMI) media (RPMI-1640; Sigma-Aldrich) with 10 % foetal bovine serum and plated in 24-well tissue culture plates (Sigma-Aldrich) at a concentration of 2 × 10^6^ cells per well. Plated PBMC were cultured with medium alone (control), with 1 multiplicity of infection human rhinovirus A16 (RV-16) or with 0·1 MOI influenza A/2009 pandemic (pdm09) (H1N1)^(31)^ for 48 h (33 °C, 5 % CO_2_). Cell suspensions were centrifuged at ∼550 × ***g*** for 5 min at 4°C, and cell-free supernatant was stored at −80 °C for analysis. The concentrations of IFN-γ, IFN-α2, TNF-α and IL-6 in the culture supernatants were analysed using the bead-based multiplex assay LEGENDPlex™ (BioLegend) as per the manufacturer’s recommendations^(32)^. The range of standards for the assay was 2·2 – 9000 pg/mL for IFN-γ, 3·2–13 000 pg/mL for IFN-α2 and TNF-α, and 2·4 − 10 000 pg/mL for IL-6. The minimum detectable concentrations were 1·5 pg/mL for IFN-γ, 2·0 pg/mL for IFN-α2, 1·6 pg/mL for TNF-α and 1·5 pg/mL for IL-6. PBMC isolation and culture experiments were conducted by blinded trial staff members who were not involved in statistical analysis of the trial outcomes.

#### Immune cell subset frequency

Multi-parametric flow cytometric analysis was performed in whole blood using fluorescently conjugated antibodies for specific cell surface antigens and a lyse-wash procedure. Immune cells were stained in whole blood, and subsets were predefined based on specific surface markers (Supplementary Table S1, Supplementary Figure 1). After 25 min of incubation, red blood cells were lysed using BD FACS™ Lysing Solution (BD Biosciences) and washed. Samples were acquired on LSRFortessa™ X-20 flow cytometer using FACSDiva™ software (BD Biosciences)^(33)^. Data were processed using FlowJo (Version 10·8, Flow Jo LLC). Results are shown as positive cells in 10^6^ of CD45^+^ for granulocytes, 10^6^ of human leukocyte antigen^+^ for DC and 10^6^ of CD3+ cells for B cells, NK cells and T cells. Flow cytometry was conducted by blinded trial staff members who were not involved in statistical analysis of the trial outcomes.

#### Systemic inflammatory biomarkers

Plasma concentrations of IL-6 and TNF-α were analysed by high-sensitivity ELISA according to manufacturer’s instructions (Quantikine assays, R&D Systems). The assay’s sensitivity ranged from 0·2 to 10 pg/mL. High-sensitivity CRP was measured by Douglas Hanly Moir Pathology commercial laboratory by immunoturbidimetry (ARCHITECT *c*16000 analyser, Abbott, Abbott Park).

### Randomisation and data management

Participants were randomised to one of the three interventions (1:1:1 ratio) using stratified randomisation (sex – M/F and age group 50–64/≥65 years) and permuted blocks (block sizes of 6 and 9) to ensure balance over time (within blocks) and balance of treatment allocation within strata. Randomisation numbers were randomly assigned to one of the three interventions, and investigational product bottles were labelled only with unique randomisation numbers, rather than intervention assignments. The randomisation schedule was generated by an independent statistician who provided it to the trial contract manufacturer (Complementary Medicines Group) with labelling instructions. A randomisation module designed to assign randomisation numbers to participant ID numbers in a blinded fashion was developed in REDCap (Research Electronic Data Capture) by the independent statistician. The REDCap module was used to assign randomisation numbers to participants by trial staff who had no participant contact and were not involved in data collection or analysis. During the study, both the participants and the investigators were blinded to treatment allocation. At the completion of the final participant, the independent statistician revealed the intervention assignments within the REDCap randomisation module. Study data were collated and managed using REDCap electronic data capture tools hosted at the Hunter Medical Research Institute^(34,35)^.

### Statistical analysis

Based on previous studies, we hypothesised that we would observe an effect size of 0·8 sd between groups in IFN-γ release. To have 80 % power to detect a difference between groups, with a two tailed, type 1 error of *α* = 0·025, we required *n* 31 participants per group to complete the intervention.

Data were exported from REDCap and analysed using STATA 15 (StataCorp). Normality was assessed using Shapiro–Wilk tests. Baseline comparisons were analysed by Kruskal–Wallis tests for continuous skewed data, one-way analysis of variance for normally distributed data or *χ*^2^ or Fisher’s exact tests for categorical variables, as appropriate. Baseline dietary nutrient intake was analysed by multivariate linear regression, adjusted for age, sex, BMI and total energy intake using the residual method^(36)^. For primary and secondary outcomes, intention-to-treat analysis (using all available follow-up data) was conducted using appropriate statistical models according to the outcome. Immune cell cytokine responses, immune cell subset frequency and systemic inflammation outcomes were analysed using multivariate regression models. Log-transformed post (follow-up) immune variables were included as dependent variables, with treatment group as the independent variable and log-transformed pre (baseline) immune variables, age, BMI and time since most recent vaccination included as covariates in the model. The Health-Related Quality of Life Questionnaire-14 was analysed with mixed-effects models, with intervention group, time (pre, post) and the group-by-time interaction as fixed factors and participant as a random factor. Variance of residuals and multivariate normality were assessed for all models. The normality of residuals was evaluated using visual inspection of Q–Q plots and histograms. Homoscedasticity was assessed by plotting residuals against fitted values and examining the plots for evidence of non-constant variance, outliers or non-linear patterns. No major violations of model assumptions were observed following transformation of dependent variables, when necessary. Cytokine, immune cell and systemic inflammation changes within intervention groups were analysed by Wilcoxon matched-pairs signed-rank tests. In all analyses, *P* values <0·05 were considered statistically significant. Graphs were produced using GraphPad Prism 9.0 (GraphPad Software). Participant numbers for each analysis are presented in individual results tables and figure legends. Some outcome variable analyses contain reduced participant numbers due to laboratory analysis quality control tests.

## Results

### Participant flow

**Figure 1.**
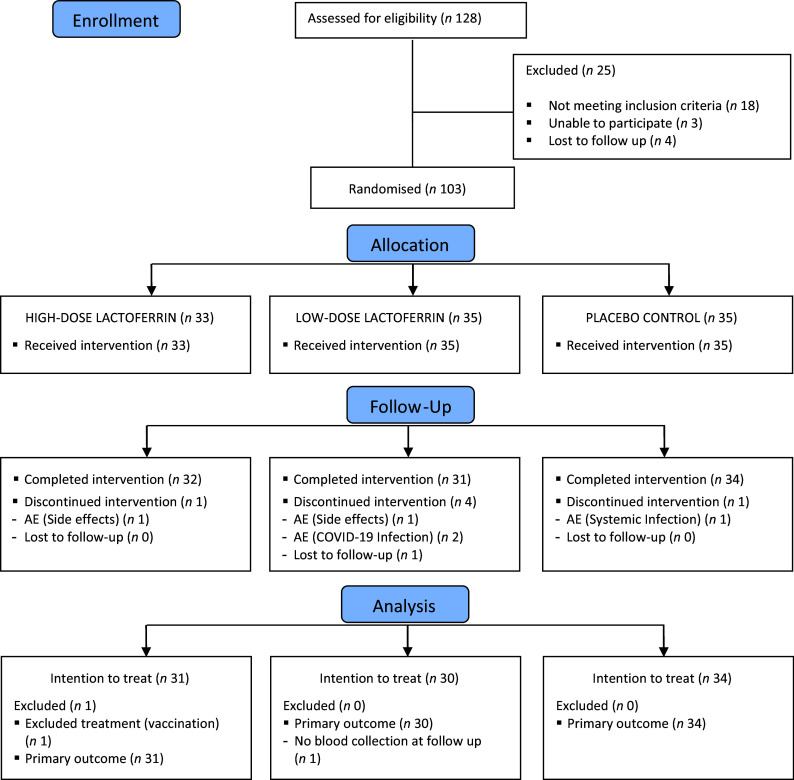
Figure 1 long description. CONSORT participant flow diagram. AE, adverse event.

Between August 2022 and September 2023, 103 participants were randomised to either the Lf-High (*n* 33) (600 mg/d Lf), Lf-Low (*n* 35) (200 mg/d Lf) or placebo (*n* 35) interventions (Figure 1). In total, ninety-seven participants completed the intervention (Lf-High *n* 32, Lf-Low *n* 31, placebo *n* 34), and six participants discontinued the intervention (Lf-High *n* 1, Lf-Low *n* 4, placebo *n* 1). Intention-to-treat analysis included 102 participants at baseline and ninety-five participants at follow-up. One participant from the Lf-High group who completed the intervention was excluded from intention-to-treat analysis due to their eligibility status changing after randomisation, for vaccine use during the intervention which was considered an excluded treatment (Figure 1).

The average adherence rates were 97 % in the Lf-High and Lf-Low groups and 100 % in the placebo group. Two participants consumed less than 80 % of the investigational product (Lf-High *n* 1, Lf-Low *n* 1) and were considered non-adherent to the intervention, with adherence rates of 52 % and 53 %, respectively.

### Participant characteristics

Participants were predominantly Caucasian Australian adults aged between 50 and 82 years. At baseline, there were no characteristic differences between intervention groups (Table 1), with similar age, sex, weight, BMI, smoking history and comorbidities between the intervention groups (Table 1). There were no differences between groups in either medication use (Supplementary Table S2), usual dietary intake (Supplementary Table S3) or the time since most recent vaccination for either influenza or COVID-19 vaccinations (Supplementary Table S4). The use of dietary or nutritional supplements was similar between groups, except for vitamin B_12_ supplement use, which was significantly different between groups, with lower use seen in the Placebo group compared with the Lf-Low group (*P* = 0·030) (Supplementary Table S2).

**Table 1.** Baseline characteristics of healthy, older adults in 4-week intervention with high (Lf-H, 600 mg/d) or low-dose (Lf-L, 200 mg/d) oral lactoferrin or placebo (PL)

	All participants		Lf-H		Lf-L		PL		P^*^
	*n* 103		*n* 33		*n* 35		*n* 35		
Demographics									
	*n*	%	*n*	%	*n*	%	*n*	%	
Sex, female	59	57	21	61	20	57	19	54	0·87
Ex-smokers	36	35	10	30	12	34	1	40	0·70
	**Mean**	**sd**	**Mean**	**sd**	**Mean**	**sd**	**Mean**	**sd**	
Age, years	64·7	8·1	63·8	7·9	64·3	8·1	66·0	8·4	0·52
Weight, kg	80·0	17·2	80·6	14·9	79·8	19·1	79·6	17·8	0·97
BMI, kg/m^2^	27·0	4·8	27·2	3·7	27·1	5·4	26·8	5·1	0·94
Smoking history, pack-years	13·9	11·7	15·4	10·0	14·7	12·9	12·1	12·5	0·77

Comorbidities									
	*n*	%	*n*	%	*n*	%	*n*	%	
Hayfever/Sinus	33	32	13	39	11	31	9	26	0·48
Respiratory	22	21	11	33	4	11	7	20	0·10
Hypertension	25	24	10	30	4	11	11	31	0·08
High cholesterol	29	28	7	21	8	23	14	40	0·16
Reflux	19	18	7	21	5	14	7	20	0·73
Diabetes	5	5	0	0	4	11	1	3	0·12
Arthritis	26	25	7	21	7	20	12	34	0·32
Psychiatric^†^	12	12	3	9	3	9	6	17	0·59

BMI, body mass index.

* Data analysed by one-way analysis of variance, Chi-squared or fisher’s exact tests.

† Depression, anxiety, attention deficit disorder.

### Effects of oral lactoferrin treatment on peripheral blood mononuclear cell cytokine expression following ex vivo stimulation with respiratory virus

#### High- and low-dose lactoferrin treatment reduces unstimulated peripheral blood mononuclear cell IL-6 expression

**Figure 2.**
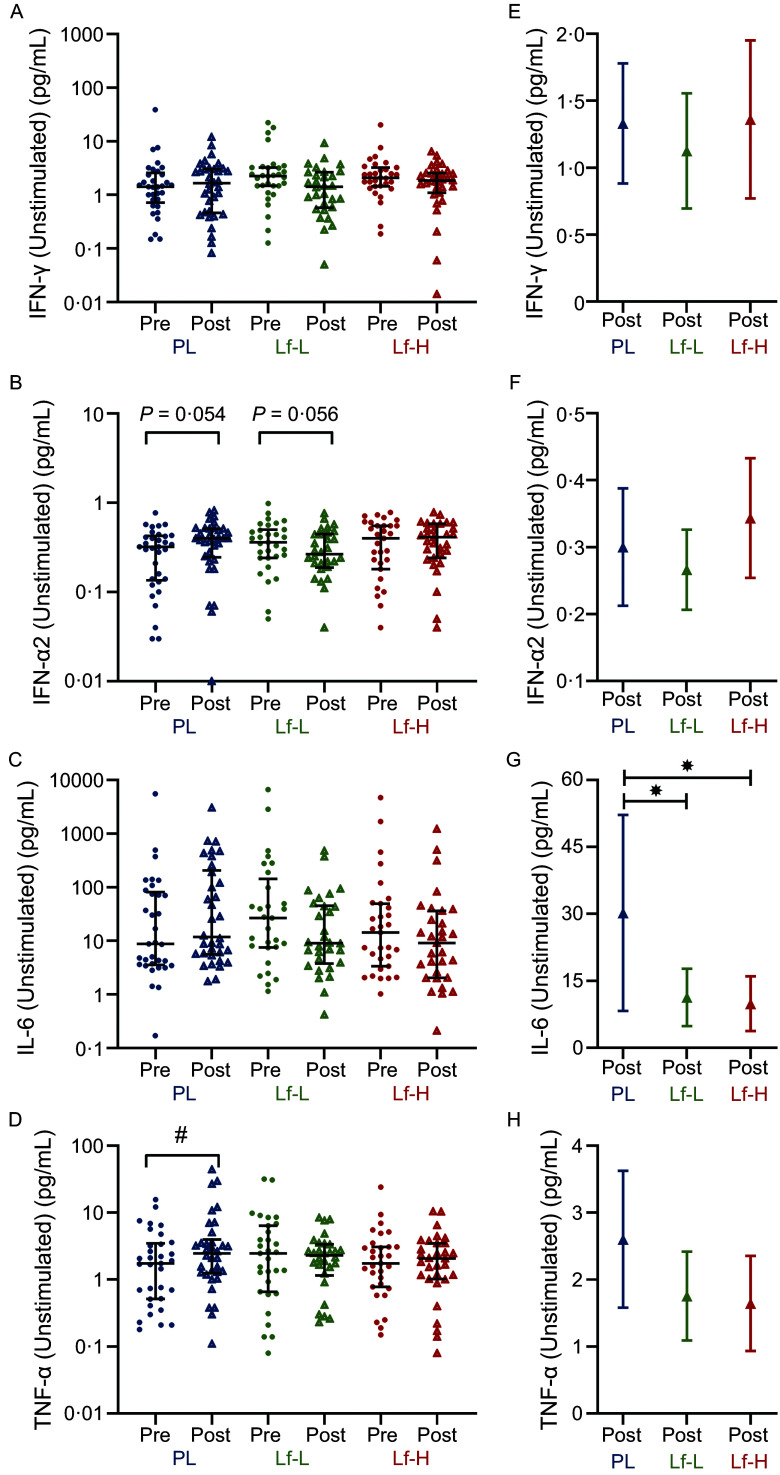
Cytokine release (pg/mL) in unstimulated PBMC from healthy, older adults at baseline (pre) and follow up (post) (unadjusted), and adjusted values at follow up (post), in 4-week intervention with high (Lf-H, 600 mg/d) or low-dose (Lf-L, 200 mg/d) oral lactoferrin or placebo (PL). **A, E:** IFN-γ (Lf-High *n* 30, Lf-Low *n* 30, PL *n* 34). **B, F:** IFN-α2 (Lf-High *n* 30, Lf-Low *n* 30, PL *n* 34). **C, G:** IL-6 (Lf-High *n* 29, Lf-Low *n* 30, PL *n* 33). **D, H:** TNF-α. (Lf-High *n* 30, Lf-Low *n* 30, PL *n* 34). A-D: pre and post data displayed as unadjusted medians with interquartile range; no differences (*P* > 0·05) in pre values between groups for all variables, analysed by Kruskal–Wallis Test; significant (#, *P* < 0·05) within-group change analysed by Wilcoxon signed-rank test. E–H: post data displayed as marginal means (95 % CI) adjusted for baseline (pre) concentration, age, BMI and time since vaccination; significant (*, *P* < 0·05) difference in post between Lf-L or Lf-H groups and placebo, or between Lf-L and Lf-H groups, analysed by multiple linear regression model adjusted for baseline (pre) concentration, age, BMI and time since vaccination. IFN-γ, interferon gamma; IFN-α2, interferon alpha-2; IL-6, interleukin-6; PBMC, peripheral blood mononuclear cell; TNF- α, tumour necrosis factor-alpha.

There was no difference in baseline (pre) unstimulated PBMC expression of IFN-γ, IFN-α, IL-6 or TNF-*α* between Lf-Low or Lf-High groups and placebo or between Lf-Low and Lf-High groups (Figure 2A–D, Supplementary Table S5). At follow-up (post), there was no difference in IFN-γ or IFN-*α*2 release between Lf-Low or Lf-High groups and placebo or between Lf-Low and Lf-High groups (Figure 2E and F). At follow-up, IL-6 release was lower in both the Lf-Low (*P* = 0·039, Figure 2G) and Lf-High groups (*P* = 0·024, Figure 2G) compared with placebo, though there was no difference in IL-6 release between Lf-Low and Lf-High groups. There was no difference in TNF-*α* release between Lf-Low or Lf-High groups and placebo or between Lf-Low and Lf-High groups at follow-up. However, when analysing within-group changes between baseline and follow-up, TNF-*α* increased in the placebo group (*P* = 0·040, Figure 2D), with no change in TNF-*α* release in the Lf-Low group or Lf-High groups.

#### High-dose lactoferrin treatment reduces rhinovirus A16-induced IL-6 expression

**Figure 3.**
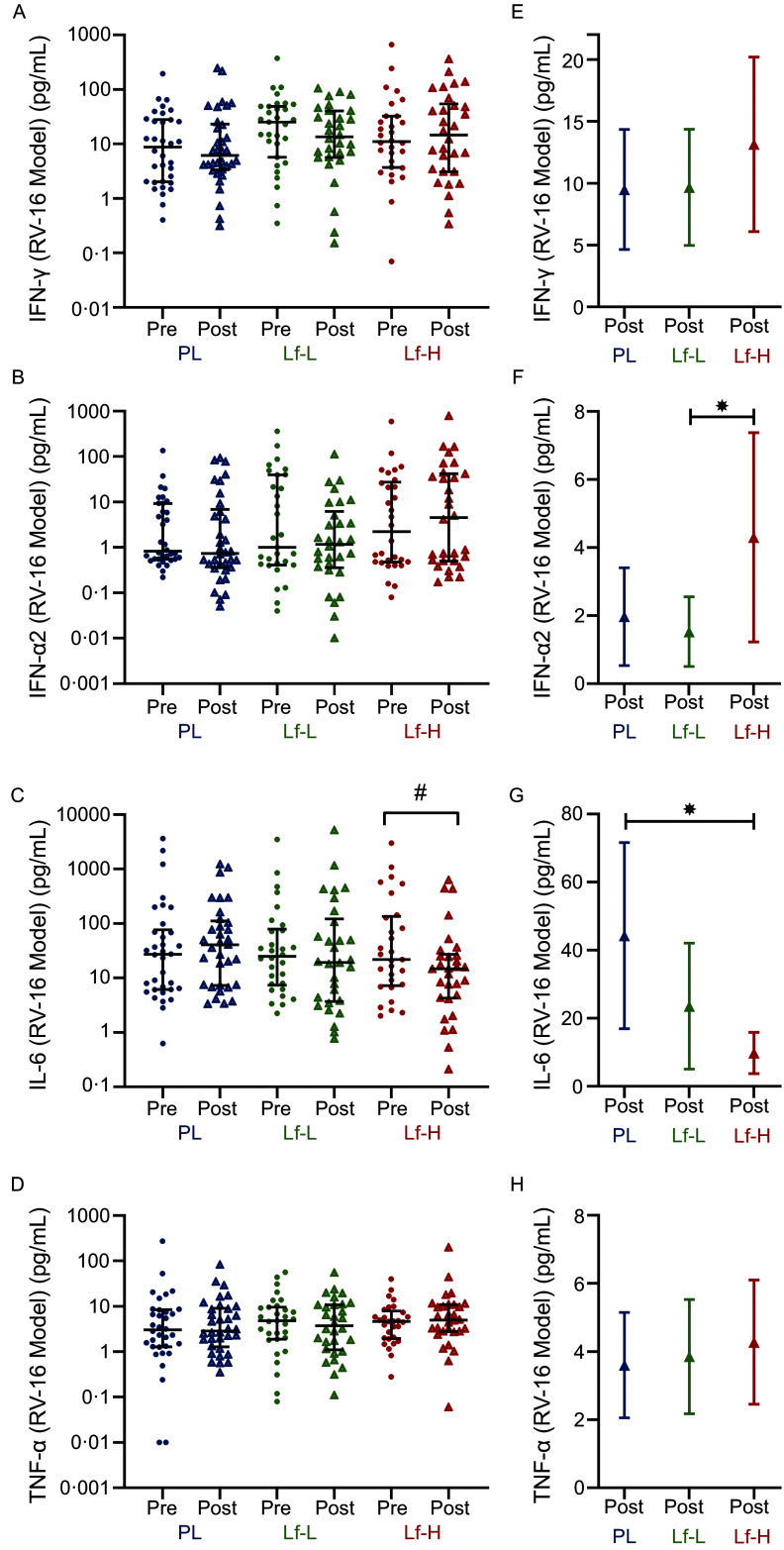
Figure 3 long description. Cytokine release (pg/mL) in rhinovirus-16 (RV-16) stimulated PBMC from healthy, older adults at baseline (pre) and follow up (post) (unadjusted), and adjusted values at follow up (post), in 4-week intervention with high (Lf-H, 600 mg/d) or low-dose (Lf-L, 200 mg/d) oral lactoferrin or placebo (PL). **A, E:** IFN-γ (Lf-High *n* 30, Lf-Low *n* 30, PL *n* 34). **B, F:** IFN-α2 (Lf-High *n* 30, Lf-Low *n* 30, PL *n* 34). **C, G:** IL-6 (Lf-High *n* 29, Lf-Low *n* 30, PL *n* 33). **D, H:** TNF-α. (Lf-High *n* 30, Lf-Low *n* 30, PL *n* 34). A-D: pre and post data displayed as unadjusted medians with interquartile range; no differences (*P* > 0·05) in pre values between groups for all variables, analysed by Kruskal–Wallis Test; significant (#, *P* < 0·05) within-group change analysed by Wilcoxon signed-rank test. E-H: post data displayed as marginal means (95 % CI) adjusted for baseline (pre) concentration, age, BMI and time since vaccination; significant (*, *P* < 0·05) difference in post between Lf-L or Lf-H groups and placebo, or between Lf-L and Lf-H groups, analysed by multiple linear regression model adjusted for baseline (pre) concentration, age, BMI and time since vaccination. IFN-γ, interferon gamma; IFN-α2, interferon alpha-2; IL-6, interleukin-6; PBMC, peripheral blood mononuclear cell; TNF- α, tumour necrosis factor-alpha.

There was no difference in baseline expression of RV-16-induced IFN-γ, IFN-*α*, IL-6 or TNF-*α* between Lf-Low or Lf-High groups and placebo or between Lf-Low and Lf-High groups (Figure 3A–D, Supplementary Table S6). At follow-up, IFN-α2 release was higher in the Lf-High group compared with the Lf-Low group (*P* = 0·041, Figure 3F), with no differences observed between placebo with Lf-Low or Lf-High groups. IL-6 release was significantly lower at follow-up in the Lf-High group compared with placebo (*P* = 0·001, Figure 3G), though there was no difference between Lf-Low with either placebo or Lf-High groups. Additionally, when analysing within-group changes between baseline and follow-up, IL-6 decreased in the Lf-High group only (*P* = 0·006, Figure 3C). There were no differences in IFN-γ or TNF-α release between Lf-Low or Lf-High groups and placebo or between Lf-Low and Lf-High groups at follow-up (Figure 3E and H).

#### High-dose lactoferrin treatment increases influenza a (H1N1)-induced TNF-α expression

**Figure 4.**
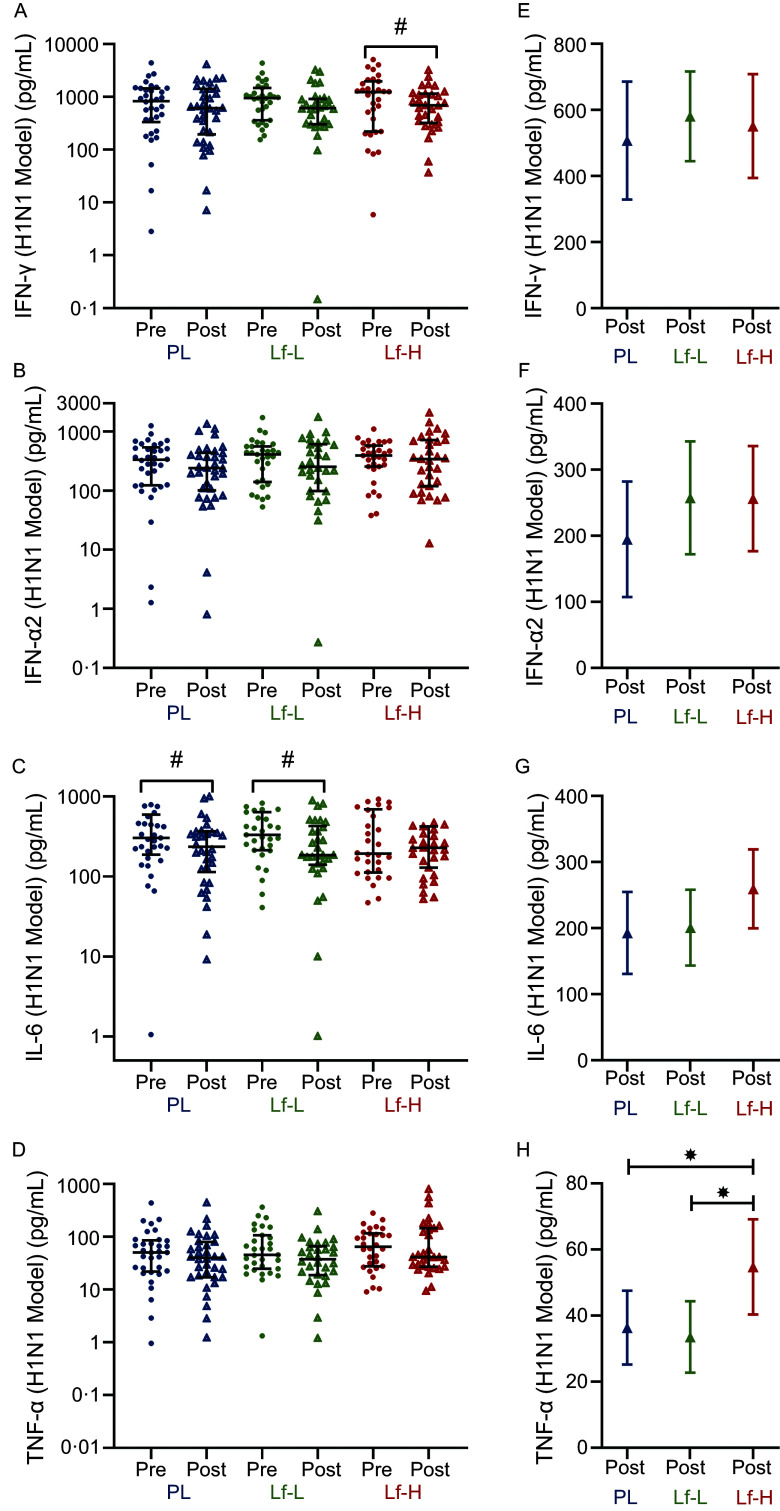
Cytokine release (pg/mL) in influenza A (H1N1) stimulated PBMC from healthy, older adults at baseline (pre) and follow up (post) (unadjusted), and adjusted values at follow up (post), in 4-week intervention with high (Lf-H, 600 mg/d) or low-dose (Lf-L, 200 mg/d) oral lactoferrin or placebo (PL). **A, E:** IFN-γ (Lf-High *n* 30, Lf-Low *n* 30, PL *n* 34). **B, F:** IFN-α2 (Lf-High *n* 31, Lf-Low *n* 30, PL *n* 34). **C, G:** IL-6 (Lf-High *n* 30, Lf-Low *n* 30, PL *n* 33). **D, H:** TNF-α. (Lf-High *n* 30, Lf-Low *n* 30, PL *n* 33). A-D: pre and post data displayed as unadjusted medians with interquartile range; no differences (*P* > 0·05) in pre values between groups for all variables, analysed by Kruskal–Wallis Test; significant (#, *P* < 0·05) within-group change analysed by Wilcoxon signed-rank test. E-H: post data displayed as marginal means (95 % CI) adjusted for baseline (pre) concentration, age, BMI and time since vaccination; significant (*, *P* < 0·05) difference in post between Lf-L or Lf-H groups and placebo, or between Lf-L and Lf-H groups, analysed by multiple linear regression model adjusted for baseline (pre) concentration, age, BMI and time since vaccination. IFN-γ, interferon gamma; IFN-α2, interferon alpha-2; IL-6, interleukin-6; PBMC, peripheral blood mononuclear cell; TNF- α, tumour necrosis factor-alpha.

There was no difference in baseline expression of H1N1-induced IFN-γ, IFN-*α*, IL-6 or TNF-*α* between Lf-Low or Lf-High groups and placebo or between Lf-Low and Lf-High groups (Figure 4A–D, Supplementary Table S7). There was no difference in IFN-γ release between Lf-Low or Lf-High groups and placebo or between Lf-Low and Lf-High groups at follow-up, though when analysing within-group changes between baseline and follow-up, IFN-γ release decreased in the Lf-High group only (*P* = 0·032, Figure 4A). There were no differences in IL-6 release between Lf-Low or Lf-High groups and placebo or between Lf-Low and Lf-High groups at follow-up, though when analysing within-group changes between baseline and follow-up IL-6 release decreased in both the Lf-Low and placebo groups (*P* = 0·009 and *P* = 0·021, respectively, Figure 4C). At follow-up, TNF-α release was higher in the Lf-High group compared with both the placebo and Lf-Low groups (*P* = 0·049 and *P* = 0·023, respectively, Figure 4H), though there was no difference between Lf-Low and placebo groups.

### High- and low-dose lactoferrin treatment modulates peripheral immune cell subsets, promoting anti-inflammatory and anti-viral systemic immunity

**Figure 5.**
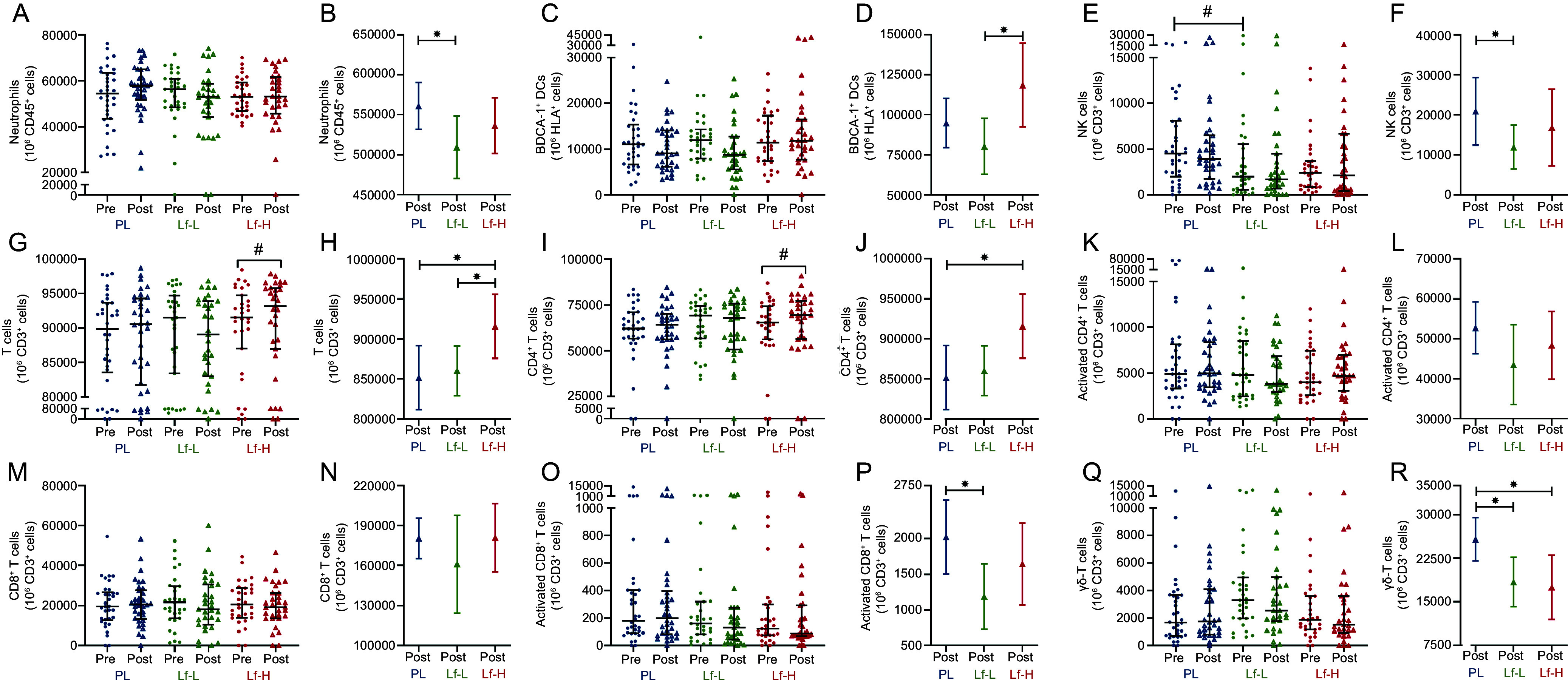
Figure 5 long description. Circulating immune cell frequency in peripheral blood from healthy, older adults at baseline (pre) and follow up (post) (unadjusted), and adjusted values at follow up (post), in 4-week intervention with high (Lf-H, 600 mg/d) or low-dose (Lf-L, 200 mg/d) oral lactoferrin or placebo (PL). **A, B**: Neutrophils. **C, D**: BDCA-1^+^ DC. **E, F:** NK cells^1^. **G, H**: T cells. **I, J:** CD4^+^ T cells. **K, L**: Activated CD4^+^ T cells. **M, N:** CD8^+^ T cells. **O, P**: Activated CD8^+^ T cells. **Q, R**: γδ-T cells. Lf-H *n* 29–30, Lf-L *n* 27–30, PL *n* 32–34. Data are displayed as unadjusted medians with interquartile range. A, C, E, G, I, K, M, O, Q: pre and post data displayed as unadjusted medians with interquartile range; difference in pre values between groups analysed by Kruskal–Wallis Test; significant (#, *P* < 0·05) within-group change analysed by Wilcoxon signed-rank test. B, D, F, H, J, L, N, P: post data displayed as marginal means (95 % CI) adjusted for baseline (pre) concentration, age, BMI and time since vaccination; significant (*, *P* < 0·05) difference in post between Lf-L or Lf-H groups and placebo, or between Lf-L and Lf-H groups, analysed by multiple linear regression model adjusted for baseline (pre) concentration, age, BMI and time since vaccination. ^1^NK cell frequency at baseline significantly higher in placebo *v*. Lf-Low group (*P* < 0·05), no other differences (*P* > 0·05) at baseline between groups. BDCA, blood dendritic cell antigen; DC, dendritic cell; NK cells, natural killer cells.

At baseline, the frequency of circulating immune cells was similar in Lf-Low and Lf-High groups compared placebo and between Lf-Low and Lf-High groups, except NK cells which were significantly higher in the placebo compared with Lf-Low group (*P* = 0·006, Figure 5E, Supplementary Table S8). At follow-up, the Lf-Low group had lower circulating neutrophils (*P* = 0·044, Figure 5B), NK cells (*P* = 0·045, Figure 5F) and activated CD8^+^ T cells (*P* = 0·031, Figure 5P), compared with the placebo, but not Lf-High group.

Both circulating total T cells and CD4^+^ T cells were significantly higher in the Lf-High group at follow-up compared with placebo (*P* = 0·031, Figure 5H & *P* = 0·028, Figure 5J, respectively) and both increased in the Lf-High group when analysing within-group changes between baseline and follow-up (*P* = 0·033, Figure 5G & *P* = 0·013, Figure 5I, respectively). Total T cell frequency was also higher (*P* = 0·037, Figure 5H) in the Lf-High group compared with the Lf-Low group at follow-up. Additionally, γδ-T cell frequency was lower following both the Lf-High (*P* = 0·046, Figure 5R) and Lf-Low (*P* = 0·031, Figure 5R) interventions compared with the placebo group. BDCA-1 myeloid DC (mDC) were higher in the Lf-High group compared with the Lf-Low group (*P* = 0·016, Figure 5D), but not placebo, with no differences seen in other DC subsets between Lf-Low or Lf-High groups and placebo or between Lf-Low and Lf-High groups (BDCA-3 mDC and pDC, Supplementary Figure S2). There were no differences between Lf-Low or Lf-High groups and placebo or between Lf-Low and Lf-High groups at follow-up in eosinophil, B cell, Treg (Supplementary Figure S2), activated CD4^+^ T cell (Figure 5L, or CD8^+^ T cell (Figure 5N), frequency.

### Systemic inflammation is reduced with high-dose lactoferrin treatment

**Figure 6.**
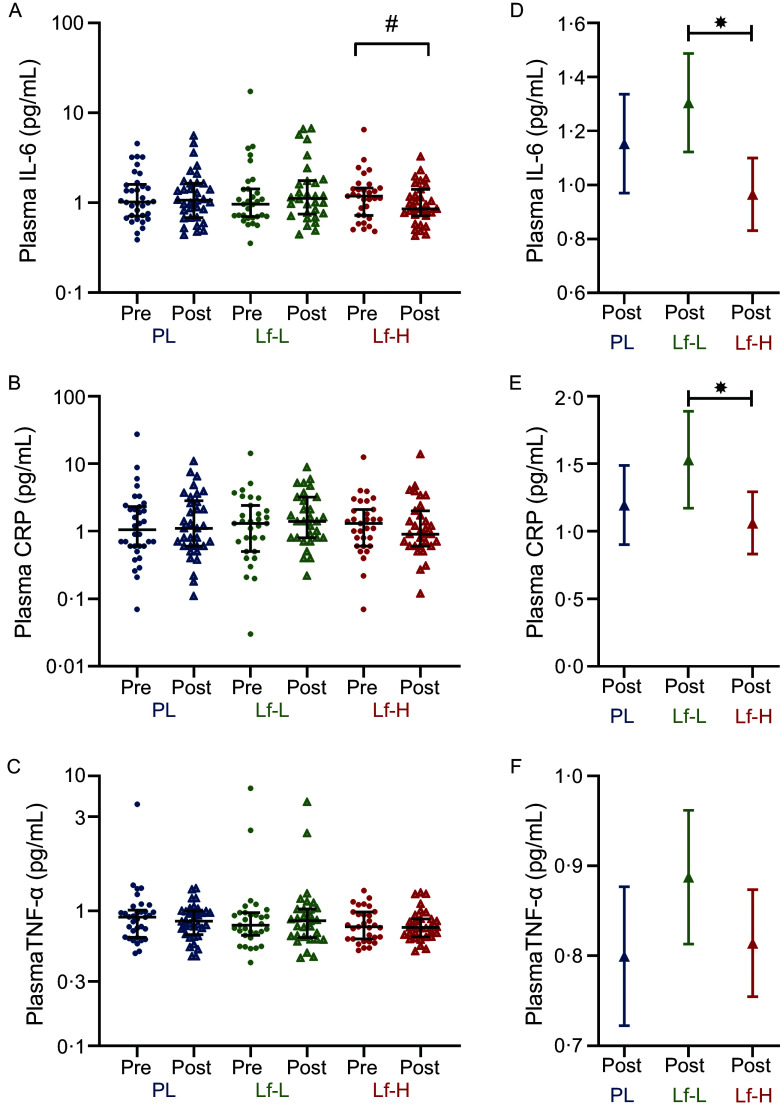
Systemic Inflammation (pg/ml) in healthy, older adults at baseline (pre) and follow up (post) (unadjusted), and adjusted values at follow up (post), in 4-week intervention with high (Lf-H, 600 mg/d) or low-dose (Lf-L, 200 mg/d) oral lactoferrin or placebo (PL). **A, D:** Plasma IL-6. **B, E**: Plasma CRP. **C, F:** Plasma TNF-α. Lf-H *n* 31, Lf-L *n* 30, PL *n* 34. A-C: pre and post data displayed as unadjusted medians with interquartile range; no differences (*P* > 0·05) in pre values between groups for all variables, analysed by Kruskal–Wallis Test; significant (#, *P* < 0·05) within-group change analysed by Wilcoxon signed-rank test. D-F: post data displayed as marginal means (95 % CI) adjusted for baseline (pre) concentration, age, BMI and time since vaccination; significant (*, *P* < 0·05) difference in post between Lf-L or Lf-H groups and placebo, or between Lf-L and Lf-H groups, analysed by multiple linear regression model adjusted for baseline (pre) concentration, age, BMI and time since vaccination. CRP, C-reactive protein; IL-6, interleukin-6; TNF- α, tumour necrosis factor-alpha.

There were no differences in baseline plasma IL-6, CRP or TNF-*α* between Lf-Low or Lf-High groups and placebo or between Lf-Low and Lf-High groups (Figure 6A–C, Supplementary Table S9). At follow-up, plasma IL-6 concentration was significantly lower in the Lf-High group compared with the Lf-Low group (*P* = 0·004, Figure 6D), but not placebo group. Plasma IL-6 significantly decreased in the Lf-High group when analysing within-group change between baseline and follow-up (*P* = 0·046) (Figure 6A). Plasma CRP concentration was also significantly lower in the Lf-High group compared with the Lf-Low group (*P* = 0·026, Figure 6E) at follow-up but not compared with placebo. There were no differences in plasma TNF-α concentration at follow-up between Lf-Low or Lf-High groups and placebo or between Lf-Low and Lf-High groups (Figure 6F).

### Health-related quality of life

There were no significant effects on participants’ general health rating or the number of healthy or unhealthy days in the previous 30 days (Supplementary Tables 10A and 10B). The number of days poor physical or mental health kept participants from participating in usual activity (impact on activity days) was higher in the Lf-High group compared with the both the placebo and Lf-Low groups (*P* = 0·003, *P* = 0·003, respectively, Supplementary Table 10A). There were no significant effects on the number of days participants experienced pain, sadness, anxiety or sleep problems. However, the number of days participants felt very healthy and full of energy (energetic days) increased in the placebo group (*P* = 0·045, Supplementary Table 10B), with this difference being significantly different to the change from baseline to follow-up in the Lf-High group (*P* = 0·045, Supplementary Table 10B).

### Adverse events

During the 4-week intervention period, a total of 134 adverse events were reported, with no difference in the total number of events or proportion of participants reporting adverse events between intervention groups for all reported adverse events (Table 2). One participant each from the Lf-High and Lf-Low groups withdrew from the trial due to intervention-related adverse events.

**Table 2.** Frequency and severity of adverse events reported by healthy, older adults in 4-week intervention with high (Lf-H, 600 mg/d) or low-dose (Lf-L, 200 mg/d) oral lactoferrin or placebo (PL) Table 2 long description.

	All participants *n* 103		Lf-H *n* 33		Lf-L *n* 35		PL *n* 35		P^*^
	*n*	%	*n*	%	*n*	%	*n*	%	
Total events reported	134	100	38	28	53	40	45	36	0·69
Participants with AE,	67	65	19	58	23	66	25	71	0·49
AEs /Participant, med (IQR)	2	1, 2·5	2	1, 3	2	1, 3	1	1, 2	0·64
Serious AE, *n*	1		0		0		1		0·61
Headache events, *n*	35		9		12		14		0·74
Participants with AE,	26	25	7	21	10	29	9	26	0·78
Severity^†^	22/10/3		6/3/0		5/4/3		11/3/0		0·16
Joint pain events, *n*	10		2		2		6		0·26
Participants with AE,	7	7	2	6	2	6	5	14	0·43
Severity^†^	5/5/0		1/1/0		0/2/0		4/2/0		0·68
URTI events, *n*	17		5		6		6		0·98
Participants with AE,	16	16	5	15	5	14	6	17	0·94
Severity^2^	12/5/0		3/2/0		4/2/0		5/1/0		0·82
Nausea events, *n*	6		2		2		2		0·10
Participants with AE,	6	6	2	6	2	6	2	6	0·99
Severity^†^	4/1/1		2/0/0		1/0/1		1/1/0		1·00
Loose Stools events, *n*	21		6		8		7		0·93
Participants with AE,	15	15	5	15	5	14	5	14	0·99
Severity^†^	15/5/1		4/1/1		7/1/0		4/3/0		0·41
GIT Pain events, *n*	12		2		7		3		0·24
Participants with AE,	10	10	2	6	6	17	2	6	0·19
Severity^†^	5/5/2		0/2/0		4/1/2		1/2/0		0·24
Flatulence events, *n*	4		2		2		0		0·36
Participants with AE,	4	4	2	6	2	6	0		0·34
Severity^†^	2/1/1		0/1/1		2/0/0				0·33
Fatigue events, *n*	4		2		2		0		0·36
Participants with AE,	3	3	1	3	2	6	0		0·38
Severity^†^ *n*	1/1/2		0/0/2		1/1/0				0·33

PL, placebo; AE, adverse event; URTI, upper respiratory tract infection.

* *P* values for the difference between intervention groups in number of participants with AE and severity of AE calculated using *X*^2^ test or Fisher’s exact test, *P* values for the difference between intervention groups in AE frequency per participant calculated using negative binomial regression.

† Severity: Mild/Moderate/Severe.

## Discussion

This trial investigated the immunomodulatory effects of high- and low-dose oral Lf supplementation for 4 weeks, in healthy, older adults. Both 600 mg/day (Lf-High, high dose) and 200 mg/day (Lf-Low, low dose) Lf interventions modulated *ex vivo* immune cell responses, with decreased pro-inflammatory cytokine and increased anti-viral cytokine release seen in response to RV-16 stimulation following the Lf-High intervention. Both doses modified circulating immune cell subsets, with increased frequency of T-cell subsets following Lf-High intervention and decreased innate immune cell frequency following Lf-Low. Additionally, systemic inflammation was reduced following the Lf-High intervention, with lower IL-6 and CRP seen in comparison to the Lf-Low intervention. Many of the immunomodulatory effects were only observed following Lf-High, and some outcomes indicated superior effects with the Lf-High intervention, in comparison with the Lf-Low intervention. These results indicate that high-dose Lf supplementation modifies immune cell responses to virus infection and decreases markers of systemic inflammation, while effects on peripheral immune cell subset frequency appear to be modulated differentially by Lf dose.

Cytokine production in unstimulated PBMC highlighted the effects of oral Lf on immune cell activity. Both Lf-High and Lf-Low interventions were associated with decreased production of the pro-inflammatory cytokine IL-6. These results suggest reduced pro-inflammatory activation in PBMC, which may indirectly improve interferon production and anti-viral immunity^(37)^.

High-dose Lf appeared superior in the context of RV-16 stimulation, as the Lf-High intervention reduced *ex vivo* PBMC IL-6 release and concurrently increased IFN-α2 release. In the context of influenza A (H1N1) stimulation, the Lf-Low intervention decreased IL-6 release, while the Lf-High intervention reduced IFN-γ release. IFN-α2 and IFN-γ are type I and type II interferons that are expressed by immune cells in response to viruses, including respiratory viruses^(38)^. These cytokines are well documented in the literature as having clear anti-viral properties by inhibiting viral replication^(39,40)^. Pro-inflammatory cytokines IL-6 and TNF-α both have important roles in viral immunity as complete knockout of these cytokines is detrimental to the host in viral infection^(41,42)^; however, over production of these cytokines is also associated with more severe lung pathology and respiratory failure in respiratory virus infections^(42,43)^.

Previous studies have also shown that oral Lf supplementation modulates *ex vivo* immune cell responses; however, these studies have been limited to stimulation with lipopolysaccharide or toll-like receptor (TLR) agonists rather than with live virus^(22,44,45)^. Similar to the results reported here, Ishikado et al.^(22)^ reported that a 4-week oral Lf (180 mg/day) intervention in periodontal disease decreased lipopolysaccharide-stimulated, PBMC release of IL-6, TNF-α, IL-1β and monocyte chemoattractant protein-1. Other studies have investigated the effects of oral Lf on peripheral blood pDC response to a TLR7/8 agonist^(44,45)^. TLR7 and TLR8 are both pattern-recognition receptors, which detect viral nucleic acids and initiate anti-viral responses^(46)^. Miyakawa et al.^(45)^ reported that Lf supplementation of 200 mg/day for 4 weeks increased TLR7/8 induced IFN-α production in peripheral blood pDC compared with the placebo group, while Van Splunter et al.^(44)^ found that a 3-week Lf (1 g/day) intervention tended to increase IFN-α production from TLR7/8 stimulated pDC. In the current study, the higher levels of IFN-α released in response to RV-16 in Lf-High *v*. Lf-Low is analogous to the IFN-α response of TLR7/8 stimulated pDC in previous studies^(44,45)^. The mechanism of action suggested by *in vitro* studies is that in the presence of a TLR7/8 agonist, Lf is taken up by pDC via both phagocytosis and endocytosis, increases pDC cell surface activation markers (HLA-DR, CD86) and IFN-α production by stimulating interferon regulatory factor and therefore IFN-sensitive response element activation^(47)^. Similar reports in the literature and observations from the current study support the hypothesis that Lf supplementation may improve anti-viral and decrease pro-inflammatory responses of PBMC to various stimuli, including viruses and bacteria.

This study also highlights the effects of oral Lf on peripheral immune cell profiles. Lf-High and Lf-Low interventions appeared to affect different immune cell subsets, apart from γδ-T cells which were reduced by both doses, and a superior effect by dose was only seen in total T-cells following Lf-High. While their roles in immunity are complex, the innate immune cells reduced by Lf supplementation (neutrophils, NK cells and γδ-T cells) were pro-inflammatory, further these cells and CD8^+^ T cells have cytotoxic activity^(48)^. A consistent finding in the literature is that Lf supplementation enhances the functions of innate and cytotoxic cells, thus in the present study, the lower abundance of these cells seen following the Lf-Low intervention may reflect increased efficiency of immune responses, necessitating fewer cell numbers. In support of this hypothesis, a 3-month oral Lf intervention (300 mg/day) increased neutrophil phagocytic capacity and NK cell cytotoxicity^(49)^, while 1·5 g and 3 g Lf daily for 12 months also increased NK cell activity^(50)^.

The Lf-High intervention increased BDCA-1 DC, the predominant circulating subset of mDC, which are potent stimulators of naïve T cells^(51)^, and CD4^+^ T cells. This increase was seen in tandem with higher total and CD4^+^ T cells. Previously, low-dose Lf (≤300 mg) had no effect on either CD4^+^ or CD8^+^ T cell frequency after 1–2 weeks^(17)^, or 3 months^(49)^, though a 7-day Lf intervention (100 mg/d and 200 mg/d) reported increased activation of both CD4^+^ and CD8^+^ T cells following both doses^(17)^. *In vitro* studies suggest that Lf promotes DC maturation, shifting away from IL-6 production and promoting the priming of naïve T cells^(52)^, and increases the expression of pDC activation markers in healthy adults after 12 weeks (200 mg/d)^(53)^. Lf treatment has also been shown to increase CD4^+^ T cell frequency in both colorectal polyps of adult humans^(50)^, and in the small intestine^(54)^ and oral tumours in mice^(55)^. These findings demonstrate that the increased CD4^+^ T cells induced by the Lf-High intervention may beneficially influence tissue immunity in the context of disease. *In vitro* studies^(47,56–58)^ show that circulating immune cells are only activated by Lf in the presence of an external stimulus^(57)^. However, while NK and pDC cell activation was not assessed in this study, previous oral Lf interventions^(49,50)^, and the changes in immune cell profiles and cytokine production in unstimulated PBMC seen in this trial, indicate that immunomodulatory effects can occur in the absence of an infectious or inflammatory stimulus. Overall, the effects of Lf supplementation on peripheral immune cell populations indicate a shift away from innate and cytotoxic immunity that can contribute to pathology in disease, towards the promotion of adequate cellular adaptive immune responses mediated through CD4^+^ cells, which provide protection against infection, tumours and chronic disease. Future work could consider assessing immune cell activity, in addition to abundance and response to infection following oral supplementation.

Only high-dose Lf decreased systemic inflammation in this study, with lower IL-6 and CRP seen in Lf-High compared with Lf-Low, though concentrations were not different to the placebo group. Other trials have shown that lower Lf doses are able to reduce IL-6, with doses ≤200 mg/d or equivalent to the Lf-Low intervention^(14,23,25,44,59,60)^, though these studies were mostly conducted in populations with inflammatory diseases or disrupted iron homeostasis. The literature does not show consistent effects of Lf supplementation on circulating CRP. Longer duration trials with doses ≤200 mg/d for 12 weeks have reported decreased CRP levels, though other trials with both higher and lower doses than the high-dose intervention have shown no effect^(59,61)^. Plasma TNF-α was not affected by either the Lf-High or Lf-Low intervention in this study. Other studies with doses ≤200 mg/d and longer duration (3 months) have shown reductions in plasma TNF-α^(59,61)^, while a trial with 1000 mg/d for 12 weeks showed no effect on this biomarker^(62)^. In healthy older adults, a higher dose of Lf may be required to reduce systemic inflammatory biomarkers, though further evidence is required in this population group.

To provide additional context to our results, PBMC are a large component of the inflammatory cells present in whole blood, which remain after removal of red blood cells and granulocytes (neutrophils, basophils and eosinophils)^(20)^. PBMC are comprised mostly of lymphocytes, including T cells, B cells and NK cells, as well as monocytes and lower numbers of DC^(63)^. They are the key drivers of immune responses, undergoing activation, proliferation and differentiation into many immune cell subsets^(64)^. Importantly, *in vivo* responses are not limited to one cell type, and primary human PBMC culture models can be used to study both the innate and adaptive immune response to infection and the interplay between them, if interpreted within the context of *in vivo* observations^(65)^. For example, Lf-High did not alter the abundance of pDC, but increased RV-16-stimulated release of IFN-α2 from PBMC. Given that pDC^(66)^ are the major cellular source of IFN-α, this suggests that phenotypic changes, and not immune cell proliferation, mediate anti-viral effects of Lf. Similarly, lower IL-6 responses from unstimulated and RV-16-stimulated PBMC are consistent with reduced serum IL-6 levels seen following Lf-High and may indicate a shift to a mature DC phenotype which promotes T cell proliferation^(52)^, which is consistent with increased abundance of T cells observed after LF-High. Many T cell subtypes produce TNF-α^(67)^, which may explain why H1N1-stimulated PBMC increased TNF-α production after Lf-High, but not after Lf-Low which reduced activated CD8^+^ T cells, and NK cells, another producer of TNF-α^(68)^. Further understanding of these interactions will require investigation of the interactions of T cell subtypes, DC and other antigen-presenting cells and their impact on intracellular signalling pathways and cytokine production following Lf treatment.

The immunomodulatory effects seen in this trial indicate that Lf supplements, particularly in higher doses, may be beneficial in the context of respiratory tract infections in older adults. Previous trials investigating respiratory tract infection prevention, or symptom amelioration (using different populations, dosages, durations and formulations), present mixed results. A recent systematic review found that 50 % of studies (*n* 5/10) reported benefits of Lf supplementation, specifically in reducing the frequency or duration of RTI in interventions of at least 3 months and up to 12 months, with reduced incidence seen mostly in 12-month interventions in infants^(19)^. Additionally, some studies have examined whether Lf supplementation could specifically prevent SARS-CoV-2 infection or ameliorate COVID-19 disease (*recently reviewed in*^(69)^); however, the evidence to date does not support its use in this condition. Well-conducted randomised controlled trials indicate that oral Lf (600 mg/d) had no effect on the incidence or clinical outcomes of COVID-19 in health care workers after supplementation for 3 months^(70)^, or clinical outcomes in hospitalised patients with moderate-to-severe disease, after treatment with 800 mg/d for 1 month after infection^(71)^. Other studies that were not randomised and/or placebo controlled reported either nil^(72,73)^ or some^(74)^ benefit in symptom resolution after treatment, with varied doses ranging from 100 mg/d to 1000 mg/d for periods of 7–42 days, though indicated that conversion time to SARS-CoV-2 RNA seronegative may be reduced with 200 mg/d to 1000 mg/d Lf treatment for 7–30 days^(73,75)^. While the current 4-week intervention showed positive effects on ex vivo immune cell responses to respiratory virus stimulation, evidence from longer duration intervention trials with RTI surveillance in older adults would be helpful in determining response to infection in vivo.

This study was performed according to rigorous methodology, with double-blinded, random allocation and appropriate masking. Primary outcomes were objectively measured in biospecimens and analysed according to intention to treat principles, reducing the likelihood of bias. A limitation of the current study is that the activity of some immune cell subsets (neutrophils and NK cells) was not assessed. Additionally, while the intervention was generally well tolerated, and attempts were made to minimise loss to follow-up, some participants withdrew due to adverse events, thus did not have complete data for follow-up assessments. These withdrawals, combined with the exclusion of individual participant data due to laboratory quality control standards, resulted in a reduced sample size in both the Lf-High and Lf-Low groups. Consequently, some analyses may have been underpowered relative to the a priori sample size calculation. As participants were permitted to consume dietary and nutritional supplements (only if taken daily, excluding Lf) during the trial, some participants (Lf-High *n* 2, Lf-Low *n* 1) were using probiotic supplements. The use of probiotics by some participants may have affected individual results, as oral Lf has been shown to act synergistically with certain probiotics in the context of gastrointestinal infection^(76)^. Finally, the quantities of *Beta vulgaris* root powder^(77)^, maltodextrin^(78)^ and microcrystalline cellulose^(79)^ used as excipients in the manufacture of the intervention treatments are considered to be inert and unlikely to exert immunomodulatory effects.

In conclusion, a 4-week intervention in healthy, older adults showed that a high-dose Lf supplement enhanced *ex vivo* immune cell responses to virus infection, while both high- and low-dose supplementation positively modulated immune cell frequency and activity in peripheral blood. High-dose Lf increased T-cell subsets, promoting adaptive immunity, while low-dose Lf reduced the frequency of proinflammatory and cytotoxic immune cells. However, beneficial effects on systemic inflammation were only seen following high-dose Lf supplementation. Oral Lf supplements are generally regarded as safe and appear to have immunoceutical benefits in healthy, older adults.

## Supporting information

Berthon et al. supplementary material 1 Berthon et al. supplementary material

Berthon et al. supplementary material 2 Berthon et al. supplementary material

## Supplementary material

To view supplementary material for this article, please visit https://doi.org/10.1017/S000711452610631X

## Figure 1 Long description

A flowchart illustrating the stages of a clinical trial involving lactoferrin and placebo control groups. The flowchart begins with the enrollment phase where 128 participants were assessed for eligibility. 25 participants were excluded for various reasons, including not meeting inclusion criteria, inability to participate, and loss to follow-up. 103 participants were randomized into three groups: high-dose lactoferrin, low-dose lactoferrin, and placebo control. Each group received the respective intervention. During the follow-up phase, participants either completed the intervention or discontinued it due to adverse events or other reasons. In the high-dose lactoferrin group, 32 completed the intervention, 1 discontinued due to side effects, and none were lost to follow-up. In the low-dose lactoferrin group, 31 completed the intervention, 4 discontinued due to side effects or COVID-19 infection, and 1 was lost to follow-up. In the placebo control group, 34 completed the intervention, 1 discontinued due to systemic infection, and none were lost to follow-up. The analysis phase includes the intention to treat and primary outcome for each group.

Navigate back to Figure 1.

## Figure 3 Long description

The image contains eight scatter plots and four bar graphs showing cytokine release levels in healthy, older adults before and after a 4-week intervention with lactoferrin or placebo. Panel A: A scatter plot displays IFN-gamma levels (pg/mL) before and after the intervention for placebo (PL), low-dose lactoferrin (Lf-L), and high-dose lactoferrin (Lf-H) groups. The y-axis ranges from 0.01 to 1000 pg/mL. Panel B: A scatter plot shows IFN-alpha2 levels (pg/mL) with the same group labels and y-axis range as Panel A. Panel C: A scatter plot depicts IL-6 levels (pg/mL) with the same group labels and y-axis range as Panel A. Panel D: A scatter plot illustrates TNF-alpha levels (pg/mL) with the same group labels and y-axis range as Panel A. Panel E: A bar graph presents adjusted IFN-gamma levels (pg/mL) after the intervention for the same groups, with the y-axis ranging from 0 to 20 pg/mL. Panel F: A bar graph shows adjusted IFN-alpha2 levels (pg/mL) with the same group labels and y-axis range as Panel E. Panel G: A bar graph depicts adjusted IL-6 levels (pg/mL) with the same group labels and y-axis range as Panel E. Panel H: A bar graph illustrates adjusted TNF-alpha levels (pg/mL) with the same group labels and y-axis range as Panel E. The scatter plots show unadjusted median values with interquartile ranges, while the bar graphs display marginal means with 95% confidence intervals. Significant differences within groups and between groups are indicated by symbols.

Navigate back to Figure 3.

## Figure 5 Long description

The image contains multiple scatter plots with error bars and box plots showing the frequency of various immune cells in peripheral blood from healthy, older adults at baseline (pre) and follow-up (post) after a 4-week intervention with high-dose (Lf-H, 600 mg/d) or low-dose (Lf-L, 200 mg/d) oral lactoferrin or placebo (PL). Each panel represents different types of immune cells. Panel A, C, E, G, I, K, M, O, Q: Pre and post data displayed as unadjusted medians with interquartile range. Panel B, D, F, H, J, L, N, P: Post data displayed as marginal means with 95 percent confidence intervals adjusted for baseline concentration, age, body mass index, and time since vaccination. Panel A and B: Neutrophils. Panel C and D: BDCA-1+ dendritic cells. Panel E and F: Natural killer cells. Panel G and H: T cells. Panel I and J: CD4+ T cells. Panel K and L: Activated CD4+ T cells. Panel M and N: CD8+ T cells. Panel O and P: Activated CD8+ T cells. Panel Q and R: gamma delta T cells. The x-axis represents the different groups (PL, Lf-L, Lf-H) and the time points (pre and post). The y-axis represents the frequency of the respective immune cells. Significant differences within groups are analyzed by the Wilcoxon signed-rank test, and significant differences between groups are analyzed by a multiple linear regression model adjusted for baseline concentration, age, body mass index, and time since vaccination.

Navigate back to Figure 5.

## Table 2 Long description

A table comparing the frequency and severity of adverse events reported by healthy, older adults in a 4-week intervention with high or low-dose oral lactoferrin or placebo. The table has 103 participants divided into three groups: Lf-H (33 participants), Lf-L (35 participants), and PL (35 participants). The table includes columns for all participants, Lf-H, Lf-L, and PL, with rows for total events reported, participants with adverse events (AE), AEs per participant, serious AE, headache events, participants with AE, severity, joint pain events, participants with AE, severity, upper respiratory tract infection (URTI) events, participants with AE, severity, nausea events, participants with AE, severity, loose stools events, participants with AE, severity, gastrointestinal (GIT) pain events, participants with AE, severity, flatulence events, participants with AE, severity, and fatigue events, participants with AE, severity. Row 1: Total events reported, 134, 38, 53, 40, 36. Row 2: Participants with AE, 67, 19, 23, 25, 26. Row 3: AEs per participant, med (IQR), 2, 1, 2.5, 1, 3, 2, 1, 3, 1, 2. Row 4: Serious AE, n, 1, 0, 0, 1. Row 5: Headache events, n, 35, 9, 12, 14. Row 6: Participants with AE, 26, 7, 10, 9, 26. Row 7: Severity, 22/10/3, 6/3/0, 5/4/3, 11/3/0. Row 8: Joint pain events, n, 10, 2, 2, 6. Row 9: Participants with AE, 7, 2, 2, 5, 14. Row 10: Severity, 5/5/0, 1/1/0, 0/2/0, 4/2/0, 6/0/0. Row 11: URTI events, n, 17, 5, 6, 6. Row 12: Participants with AE, 16, 5, 5, 14, 17. Row 13: Severity, 12/5/0, 3/2/0, 4/2/0, 5/1/0. Row 14: Nausea events, n, 6, 2, 2, 2. Row 15: Participants with AE, 6, 2, 2, 2, 6. Row 16: Severity, 4/1/1, 2/0/0, 1/0/1, 1/1/0. Row 17: Loose stools events, n, 21, 6, 8, 7. Row 18: Participants with AE, 15, 5, 5, 14, 14. Row 19: Severity, 15/5/1, 4/1/1, 7/1/0, 4/3/0. Row 20: GIT pain events, n, 12, 2, 7, 3. Row 21: Participants with AE, 10, 2, 6, 17, 6. Row 22: Severity, 5/5/2, 0/2/0, 4/1/2, 1/2/0. Row 23: Flatulence events, n, 4, 2, 2, 0. Row 24: Participants with AE, 4, 2, 2, 0, 6. Row 25: Severity, 2/1/1, 0/1/1, 2/0/0, 0/0/0. Row 26: Fatigue events, n, 4, 2, 2, 0. Row 27: Participants with AE, 3, 1, 2, 0, 6. Row 28: Severity, 1/1/2, 0/0/2, 1/1/0, 0/0/0.

Navigate back to Table 2.
