# Supplementary material for: Oral lactoferrin reduces systemic inflammation, enhances anti-viral responses and modulates immune cell profiles: a randomised controlled trial in healthy, older adults
Source: Br J Nutr. 2026 Feb 4;135(9):929–43. doi: 10.1017/S000711452610631X (PMC13315551; doi:10.1017/S000711452610631X)
Supplement: Berthon et al. supplementary material 1 — Berthon et al. supplementary material [file S000711452610631Xsup001.docx]

**Supplementary Material**

| **Table S1.** Whole Blood Flow Cytometry: Surface markers used to identify immune cell subsets in whole blood from healthy, older adults in 4-week oral lactoferrin intervention. | |
| --- | --- |
| **Cell type** | **Surface markers** |
| Leukocytes | CD45+ |
| Granulocytes | CD45^+^ SSC ^int/high^ |
| Eosinophils | CD45^+^ SSC ^int/high^ CD193^high^ |
| Neutrophils | CD45^+^ SSC ^int/high^ CD193^low^ |
| B cells | CD3^-^ CD19^+^ |
| NK cells | CD3^-^ CD56^+^ CD16^+^ |
| BDCA-1 myeloid DCs | CD3^-^ CD19^-^ CD56^-^ CD14^-^ CD16^-^ BDCA-1^+^ |
| BDCA-3 myeloid DCs | CD3^-^ CD19^-^ CD56^-^ CD14^-^ CD16^+^ CD1c^-^ CD303^-^ BDCA-3^high^ |
| pDCs | CD3^-^ CD19^-^ CD56^-^ CD14^-^ BDCA-2^+^ |
| T cells | CD3^+^ |
| CD4 T cells | αβ TCR^+^ CD4^+^ |
| CD8 T cells | αβ TCR^+^ CD8^+^ |
| Activated CD4 T cells | αβ TCR^+^ CD4^+^ CD25^+^ CD127 ^high/+^ |
| Activated CD8 T cells | αβ TCR^+^ CD8^+^ CD25^+^ CD127 ^high/+^ |
| Treg cells | αβ TCR^+^ CD4^+^ CD25^+^ CD127 ^low/-^ |
| γδ T cells | γδ TCR^+^ CD4^+^ |
| SSC, side scatter; int, intensity; BDCA, blood dendritic cell antigen; CD: cluster of differentiation; DC, dendritic cell; NK, natural killer; TCR**,** T-cell receptor; Treg, regulatory T cells; pDCs, plasmacytoid dendritic cells. | |

| **Table S2.** Medication and supplement use at baseline in healthy^1^, older adults in 4-week intervention with high (Lf-H, 600mg/d) or low dose (Lf-L, 200mg/d) oral lactoferrin or placebo (PL). | | | | | | | |
| --- | --- | --- | --- | --- | --- | --- | --- |
|  | **All Participants**  *n*=103 | | **Lf-H**  *n*=33 | **Lf-L**  *n*=35 | | **Placebo**  *n*=35 | ***P***^2^ |
| **Medications** *n* (%) | | | | | | | |
| Reflux | 17 (17) | 6 (18) | | 4 (11) | 7 (20) | | 0.637 |
| Anti-hypertensive | 27 (26) | 10 (30) | | 5 (14) | 12 (34) | | 0.133 |
| Cholesterol lowering | 24 (23) | 5 (15) | | 6 (17) | 13 (37) | | 0.057 |
| NSAIDs | 16 (16) | 5 (15) | | 7 (20) | 4 (11) | | 0.625 |
| Anxiety/Depression | 11 (11) | 3 (9) | | 3 (9) | 5 (14) | | 0.784 |
| Anti-diabetic | 6 (6) | 2 (6) | | 3 (9) | 1 (3) | | 0.690 |
| Antiplatelet/Antithrombotic | 16 (16) | 5 (15) | | 4 (11) | 7 (20) | | 0.625 |
| **Supplements** *n* (%) | | | | | | | |
| Iron | 8 (8) | 5 (15) | | 2 (6) | 1 (3) | | 0.165 |
| Multivitamin | 8 (8) | 4 (12) | | 2 (6) | 2 (6) | | 0.588 |
| Calcium | 10 (10) | 4 (12) | | 4 (11) | 2 (6) | | 0.706 |
| Vitamin D | 23 (22) | 10 (30) | | 7 (20) | 6 (17) | | 0.394 |
| Magnesium | 26 (25) | 9 (27) | | 10 (29) | 7 (20) | | 0.675 |
| Fish oil | 16 (16) | 3 (9) | | 8 (23) | 5 (14) | | 0.304 |
| Glucosamine | 7 (7) | 2 (6) | | 2 (6) | 3 (9) | | 1.000 |
| Vitamin C | 11 (11) | 3 (9) | | 4 (11) | 4 (11) | | 1.000 |
| Vitamin B Complex | 4 (4) | 2 (6) | | 2 (6) | 0 | | 0.463 |
| Vitamin B_12_^3^ | 6 (6) | 1 (3)^ab^ | | 5 (14)^b^ | 0^a^ | | **0.030** |
| Biotin | 3 (3) | 0 | | 3 (9) | 0 | | 0.105 |
| Vitamin B_6_ | 2 (2) | 0 | | 2 (6) | 0 | | 0.327 |
| Niacin | 5 (5) | 3 (9) | | 1 (3) | 1 (3) | | 0.443 |
| Vitamin B_1_ | 1 (1) | 0 | | 1 (3) | 0 | | 1.000 |
| Curcumin | 3 (3) | 0 | | 2 (6) | 1 (3) | | 0.771 |
| Herbal misc | 6 (6) | 0 | | 3 (9) | 3 (9) | | 0.242 |
| Vitamin/Herb Complex | 3 (3) | 1 (3) | | 1 (3) | 1 (3) | | 1.000 |
| Probiotic | 3 (3) | 2 (6) | | 1 (3) | 0 | | 0.314 |
| NSAID, non-steroidal anti-inflammatory drug. ^1^No acute or serious illness ^(1)^. ^2^P values for the difference between intervention groups calculated using *X*^2^ test or Fisher’s exact test. ^3^Significant difference between treatment groups, values with the same superscript letter are not significantly different. | | | | | | | |

| **Table S3.** Usual dietary intake and daily dairy serves at baseline, in healthy, older adults in 4-week intervention with high (Lf-H, 600mg/d) or low dose (Lf-L, 200mg/d) oral lactoferrin or placebo (PL) | | | | | | | |
| --- | --- | --- | --- | --- | --- | --- | --- |
| **Food Frequency Questionnaire^1^** | | | | | | | |
| **Outcome** | | **All Participants**  *n*=101 | | **Lf-H**  *n*=32^1^ | **Lf-L**  *n*=34^1^ | **Placebo**  *n*=35 | ***P^2^*** |
| **Energy** MJ/d | | 8.6 (7.4, 10.0) | | 8.7 (7.5, 9.9) | 8.4 (6.5, 9.7) | 8.6 (8.2, 10.8) | 0.655 |
| **Energy** kJ/kg/d | | 108 (91, 141) | | 111 (89, 126) | 100 (93, 124) | 125 (85, 143) | 0.378 |
| **Protein** g/day | | 96 ± 25 | | 98 ± 22 | 91 ± 23 | 99 ± 30 | 0.300 |
| **Protein** g/kg/d | | 1.2 (1.0, 1.5) | | 1.2 (1.0, 1.4) | 1.1 (1.0, 1.2) | 1.3 (1.0, 1.5) | 0.574 |
| **Protein** %TEE | | 18 ± 3 | | 18 ± 2 | 18 ± 4 | 18 ± 3 | 0.702 |
| **Carbohydrate** g/d | | 180 (150, 209) | | 174 (153, 196) | 183 (142. 209) | 182 (151, 237) | 0.611 |
| **Carbohydrate** %TEE | | 35 ± 5 | | 35 ± 6 | 34 ± 5 | 36 ± 4 | 0.560 |
| **Fibre** g/day | | 27 ± 9 | | 27 ± 7 | 25 ± 10 | 27 ± 9 | 0.778 |
| **Fat** g/day | | 98 (79, 116) | | 98 (77, 115) | 96 (66, 108) | 104 (89, 117) | 0.578 |
| **Fat** %TEE | | 42 ± 6 | | 42 ± 6 | 42 ± 7 | 43 ± 5 | 0.728 |
| **SFA** g/d | | 32 (24, 39) | | 30 (24, 36) | 32 (22, 38) | 33 (25, 42) | 0.141 |
| **MUFA** g/d | | 42 (33, 51) | | 44 (32, 50) | 39 (30, 51) | 44 (37, 52) | 0.985 |
| **PUFA** g/d | | 17 (13, 21) | | 18 (13, 20) | 14 (12, 21) | 18 (14, 23) | 0.595 |
| **Sodium** g/d | | 2.1 (1.7, 2.7) | | 2.0 (1.7, 2.5) | 2.1 (1.5, 2.7) | 2.2 (1.8, 3.0) | 0.321 |
| **Potassium**  mg/d | | 3765 ± 1021 | | 3733 ± 841 | 3629 ± 991 | 3925 ± 1192 | 0.822 |
| **Calcium** mg/day | | 945 ± 299 | | 941 ± 270 | 903 ± 295 | 989 ± 328 | 0.824 |
| **Phosphorus** mg/d | | 1660 ± 420 | | 1692 ± 339 | 1581 ± 402 | 1707 ± 500 | 0.282 |
| **Magnesium** mg/d | | 497 (384, 573) | | 511 (386, 570) | 452 (375, 534) | 514 (393, 611) | 0.887 |
| **Iron** mg/day | | 12 ± 3 | | 12 ± 3 | 11 ± 3 | 12 ± 4 | 0.089 |
| **Zinc** mg/d | | 11 ± 3 | | 11 ± 3 | 10 ± 3 | 11 ± 3 | 0.136 |
| **Retinol** equiv./d | | 937 (739, 1224) | | 954 (751, 1133) | 884 (711, 1224) | 968 (757, 1296) | 0.918 |
| **Thiamin** mg/d | | 0.9 (0.6, 1.3) | | 0.9 (0.6, 1.3) | 0.7 (0.5, 1.3) | 1.1 (0.8, 1.6) | 0.657 |
| **Riboflavin** mg/d | | 0.9 (0.7, 1.4) | | 0.9 (0.7, 1.2) | 0.9 (0.6, 1.2) | 1.1 (0.7, 1.7) | 0.183 |
| **Niacin** equiv./d | | 54 (43, 65) | | 54 (43, 62) | 52 (41, 60) | 56 (42, 75) | 0.482 |
| **Vitamin C** mg/d | | 115 (84, 144) | | 111 (94, 136) | 113 (81, 153) | 117 (84, 150) | 0.962 |
| **Folic acid DFE** ug/d | | 474 (361, 627) | | 474 (395, 581) | 413 (341, 671) | 531 (361, 669) | 0.556 |
| **Vitamin D** ug/d | | 4.6 (2.6, 5.9) | | 4.1 (2.6, 6.6) | 3.9 (2.5, 6.3) | 4.8 (3.2, 5.4) | 0.934 |
| **24-Hour Food Recall** | | | | | | | |
|  | **All Participants**  *n*=103 | | | **Lf-H**  *n*=33 | **Lf-L**  *n*=35 | **Placebo**  *n*=35 | ***P^2^*** |
| **Dairy intake** Serves/day | | | 2.0 (1.5, 3.0) | 2.0 (1.5, 3.0) | 1.9 (1.0, 2.6) | 2.5 (1.5, 3.5) | 0.114 |
| **Milk intake** Serves/day | | | 1.0 (0.5, 1.8) | 1.1 (0.5, 1.6) | 1.0 (0.2, 1.5) | 1.0 (0.5, 2.0) | 0.482 |
| MJ, megajoules. d, day. kJ, kilojoules. TEE, total estimated energy intake. SFA, saturated fatty acids. MUFA, monosaturated fatty acids. PUFA, polyunsaturated fatty acids. DFE, dietary folate equivalents.  Data presented as mean±SD or median (IQR). ^1^Includes all participants at baseline who completed the food frequency questionnaire. The analysis is missing data from participants in both the Lf-H (n=1) and Lf-L (n=1) group who did not complete the questionnaire. ^2^Data analysed using One-way Analysis of Variance or Kruskal-Wallis Test as appropriate, adjusted for total energy intake using the residual method. | | | | | | | |

| **Table S4.** Vaccination history at baseline, in healthy, older adults in 4-week intervention with high (Lf-H, 600mg/d) or low dose (Lf-L, 200mg/d) oral lactoferrin or placebo (PL) | | | | | |
| --- | --- | --- | --- | --- | --- |
|  | **All Participants**  *n*=103 | **Lf-H**  *n*=33 | **Lf-L**  *n*=35 | **Placebo**  *n*=35 | ***P^1^*** |
| **Covid-19 Vaccine** |  |  |  |  |  |
| Within 30-120 days *n* (%) | 34 (33) | 12 (36) | 10 (29) | 12 (35) | 0.759 |
| Days since last Vaccine**^2^** | 178 (78, 286) | 170 (66, 250) | 181 (118, 335) | 211 (77, 297) | 0.403 |
| **Influenza Vaccine** |  |  |  |  |  |
| Within 30-120 days *n* (%) | 38 (37) | 14 (42) | 12 (34) | 12 (34) | 0.727 |
| Days since last Vaccine**^2^** | 158 (89, 306) | 155 (89, 275) | 168 (77, 321) | 158 (105, 306) | 0.733 |
| ^1^Data analysed using Chi Squared Test or Kruskal-Wallis Test. ^2^Data presented as median (IQR). | | | | | |

| **Table S5.** Cytokine release (pg/mL) in unstimulated PBMCs from healthy, older adults at baseline (pre) and follow up (post) (unadjusted), and adjusted values at follow up (post), in 4-week intervention with high (Lf-H, 600mg/d) or low dose (Lf-L, 200mg/d) oral lactoferrin or placebo (PL). | | | | | | | | | | | | |
| --- | --- | --- | --- | --- | --- | --- | --- | --- | --- | --- | --- | --- |
| **Outcome**  **Group** | **Pre**^1^  Median (IQR) | **Post**  Median (IQR) | ***P***^2^ | **Adjusted Post**^3^  Mean (95% CI) | |  | **Difference in Post**^4^  % (95% CI) | | ***P*** | |  |  |
| **IFN-γ** | | | | | | | | | | | | |
| **Lf-H** (*n*=31) | 2.1 (1.4, 3.2) | 1.9 (1.1, 2.6) | 0.119 | 1.4 (0.8, 2.0) | **Lf-H vs PL** | | | 2.2 (-42, 80.2) | | 0.939 | |  |
| **Lf-L** (*n*=30) | 2.2 (1.5, 3.2) | 1.4 (0.6, 2.6)^2^ | 0.113 | 1.1 (0.7, 1.6) | **Lf-L vs PL** | | | -15.4 (-49.3, 41.2) | | 0.518 | |  |
| **PL** (*n*=33) | 1.4 (0.8, 2.4) | 1.6 (0.5, 3.0) | 0.376 | 1.3 (0.9, 1.8) | **Lf-H vs Lf-L** | | | 20.8 (-32.8, 117.3) | | 0.524 | |  |
| **IFN-α2** | | | | | | | | | | | | |
| **Lf-H** (*n*=31) | 0.4 (0.2, 0.6) | 0.4 (0.2, 0.6) | 0.576 | 0.3 (0.3, 0.4) | **Lf-H vs PL** | | | 14.5 (-22.5, 69.1) | | 0.492 | |  |
| **Lf-L** (*n*=30) | 0.4 (0.2, 0.5) | 0.3 (0.2, 0.4) | 0.056 | 0.3 (0.2, 0.3) | **Lf-L vs PL** | | | -11.2 (-38.8, 28.7) | | 0.525 | |  |
| **PL** (*n*=33) | 0.3 (0.1, 0.4) | 0.4 (0.3, 0.5) | 0.054 | 0.3 (0.2, 0.4) | **Lf-H vs Lf-L** | | | 29 (-10.1, 85) | | 0.165 | |  |
| **IL-6** | | | | | | | | | | | | |
| **Lf-H** (*n*=31) | 14.4 (3.4, 49.5) | 9.1 (2, 36.3) | 0.248 | 9.9 (3.8, 16) | **Lf-H vs PL** | | | -67.3 (-87.5, -14) | | **0.024** | |  |
| **Lf-L** (*n*=29) | 26.7 (7.7, 98.8) | 9.1 (3.9, 43.9) | 0.172 | 11.3 (4.9, 17.7) | **Lf-L vs PL** | | | -62.6 (-85.3, -4.9) | | **0.039** | |  |
| **PL** (*n*=33) | 8.8 (3.6, 74.5) | 11.9 (5.7, 190.7) | 0.662 | 30.2 (8.3, 52.2) | **Lf-H vs Lf-L** | | | -12.6 (-63.1, 107) | | 0.757 | |  |
| **TNF-α** | | | | | | | | | | | | |
| **Lf-H** (*n*=31) | 1.8 (0.8, 3.1) | 2.1 (1, 3.5) | 0.837 | 1.6 (0.9, 2.4) | **Lf-H vs PL** | | | -36.8 (-65, 14.2) | | 0.127 | |  |
| **Lf-L** (*n*=30) | 2.5 (0.7, 6.4) | 2.3 (1.2, 3.3) | 0.797 | 1.8 (1.1, 2.4) | **Lf-L vs PL** | | | -32.6 (-61.3, 17.4) | | 0.161 | |  |
| **PL** (*n*=33) | 1.8 (0.5, 3.4) | 2.5 (1.3, 3.6)^2^ | 0.172 | 2.6 (1.6, 3.6) | **Lf-H vs Lf-L** | | | -6.2 (-48.1, 69.6) | | 0.830 | |  |
| PBMC, peripheral blood mononuclear cell. IQR, interquartile range. CI, confidence interval. IFN-γ, interferon gamma. IFN-α2, interferon alpha-2. IL-6, interleukin-6. TNF-α, tumour necrosis factor-alpha.  ^1^ No difference (*P*>0.05) in baseline values between intervention groups and placebo for all variables, analysed by Kruskal-Wallis Test. ^2^ Significant (*P*<0.05) within treatment group change analysed by Wilcoxon signed-rank test. ^3^Marginal means adjusted for baseline (pre) concentration, age, BMI, and time since vaccination. ^4^Model coefficients (odds ratios) of differences between groups were transformed and reported as percent [95% CI] difference in post variable between intervention groups, analysed by multiple linear regression model adjusted for baseline (pre) concentration, age, BMI and time since vaccination. | | | | | | | | | | | | |

| **Table S6.** Cytokine release (pg/mL) in rhinovirus-16 (RV-16) stimulated PBMCs from healthy, older adults at baseline (pre) and follow up (post) (unadjusted), and adjusted values at follow up (post), in 4-week intervention with high (Lf-H, 600mg/d) or low dose (Lf-L, 200mg/d) oral lactoferrin or placebo (PL) | | | | | | | | | | |
| --- | --- | --- | --- | --- | --- | --- | --- | --- | --- | --- |
| **Outcome**  **Group** | **Pre**^1^  Median (IQR) | **Post**  Median (IQR) | | ***P*^2^** | **Adjusted Post**^3^  Mean (95% CI) | | |  | **Difference in Post**^4^ | ***P*** |
|  |  |  |  |  |  |  |  |  | % (95% CI) |  |
| **IFN-γ** | | | | | | | | | | |
| **Lf-H** (*n*=30) | 11.0 (3.8, 32.2) | 14.7 (3.1, 49.7) | 0.766 | | | 13.1 (6.1, 20.2) | **Lf-H vs PL** | | 38.5 (-34.2, 191.4) | 0.387 |
| **Lf-L** (*n*=30) | 25.2 (5.7, 49.1) | 13.5 (5.7, 40.4) | 0.131 | | | 9.7 (5.0, 14.4) | **Lf-L vs PL** | | 1.9 (-50.2, 108.4) | 0.959 |
| **PL** (*n*=34) | 8.8 (2.0, 27.9) | 6.2 (3.4, 22.3) | 0.483 | | | 9.5 (4.6, 14.3) | **Lf-H vs Lf-L** | | 35.8 (-34.8 (183.2)) | 0.408 |
| **IFN-α2** | | | | | | | | | | |
| **Lf-H** (*n*=30) | 2.2 (0.5, 26.1) | 4.6 (0.5, 41.6) | 0.299 | | | 4.3 (0.5, 3.4) | **Lf-H vs PL** | | 118 (-24.3, 528) | 0.147 |
| **Lf-L** (*n*=30) | 1.0 (0.4, 39.4) | 1.2 (0.4, 5.0) | 0.075 | | | 1.5 (0.5, 2.6) | **Lf-L vs PL** | | -22.4 (-71.9, 114.6) | 0.621 |
| **PL** (*n*=34) | 0.8 (0.6, 9.3) | 0.7 (0.4, 6.1) | 0.804 | | | 2.0 (0.5, 3.4) | **Lf-H vs Lf-L** | | 181 (4.6, 654.8) | **0.041** |
| **IL-6** | | | | | | | | | | |
| **Lf-H** (*n*=29) | 21.8 (7.6, 127.7) | 14.8 (4.3, 25.7) | **0.006** | | | 9.8 (3.8, 15.9) | **Lf-H vs PL** | | -77.8 (-90.9, -45.9) | **0.001** |
| **Lf-L** (*n*=30) | 24.9 (7.8, 74.6) | 19.4 (3.8, 106.2) | 0.974 | | | 23.6 (5.1, 42.1) | **Lf-L vs PL** | | -46.7 (-80.5. 45.5) | 0.216 |
| **PL** (*n*=33) | 27.1 (6.2, 69.9) | 41.0 (7.4, 103.1) | 0.514 | | | 44.3 (16.9, 71.6) | **Lf-H vs Lf-L** | | -58.2 (-85.2, 18.1) | 0.099 |
| **TNF-α** | | | | | | | | | | |
| **Lf-H** (*n*=30) | 4.6 (2.0, 7.3) | 5.0 (2.8, 10.8) | 0.173 | | | 4.3 (2.5, 6.1) | **Lf-H vs PL** | | 18.8 (-36.2, 121.2) | 0.584 |
| **Lf-L** (*n*=30) | 4.8 (1.9, 9.6) | 3.8 (1.1, 10.6) | 0.681 | | | 3.9 (2.2, 5.5) | **Lf-L vs PL** | | 7.0 (-42.6, 99.5) | 0.829 |
| **PL** (*n*=34) | 3.0 (1.3, 8.3) | 2.9 (1.3, 8.8) | 1.000 | | | 3.6 (2.1, 5.1) | **Lf-H vs Lf-L** | | 11.0 (-40.3, 106.4) | 0.740 |
| PBMC, peripheral blood mononuclear cell. IQR, interquartile range. CI, confidence interval. IFN-γ, interferon gamma. IFN-α2, interferon alpha-2. IL-6, interleukin-6. TNF-α, tumour necrosis factor-alpha.  ^1^ No difference (*P*>0.05) in baseline values between intervention groups and placebo for all variables, analysed by Kruskal-Wallis Test. ^2^ Significant (*P*<0.05) within treatment group change analysed by Wilcoxon signed-rank test. ^3^Marginal means adjusted for baseline (pre) concentration, age, BMI, and time since vaccination. ^4^Model coefficients (odds ratios) of differences between groups were transformed and reported as percent [95% CI] difference in post variable between intervention groups, analysed by multiple linear regression model adjusted for baseline (pre) concentration, age, BMI and time since vaccination. | | | | | | | | | | |

| **Table S7.** Cytokine release (pg/mL) in influenza A virus (H1N1) stimulated PBMCs from healthy, older adults at baseline (pre) and follow up (post) (unadjusted), and adjusted values at follow up (post), in 4-week intervention with high (Lf-H, 600mg/d) or low dose (Lf-L, 200mg/d) oral lactoferrin or placebo (PL.) | | | | | | | | | |
| --- | --- | --- | --- | --- | --- | --- | --- | --- | --- |
| **Outcome**  **Group** | **Pre**^1^  Median (IQR) | | **Post**  Median (IQR) | ***P*^2^** | **Adjusted Post**^3^  Mean (95% CI) |  | **Difference in Post**^4^  % (95% CI) | | ***P*** |
| **IFN-γ** | |  |  |  |  |  |  |  |  |
| **Lf-H** (*n*=30) | 1233.8 (220.2, 1967.7) | | 688.3 (329.0, 1143.7) | **0.032** | 551.3 (394.5, 685.8) | **Lf-H vs PL** | 8.6 (-31.4, 72.1) | 0.722 | |
| **Lf-L** (*n*=30) | 945.7 (360.5, 1474.0) | | 608.1 (303.8, 896.4) | 0.056 | 580.9 (445.2, 716.6) | **Lf-L vs PL** | 14.5 (-25.5, 75.9) | 0.533 | |
| **PL** (*n*=34) | 827.2 (369.8, 1445.7) | | 607.0 (123.0, 1405.6) | 0.293 | 507.5 (329.1, 685.8) | **Lf-H vs Lf-L** | -5.1 (-4.8, 38.2) | 0.782 | |
| **IFN-α2** | |  |  |  |  |  |  |  |  |
| **Lf-H** (*n*=31) | 393.7 (255.0, 577.0) | | 342.0 (119.1, 714.7) | 0.754 | 256.4 (177, 335.9) | **Lf-H vs PL** | 31.5 (-24.9, 130.5) | 0.334 | |
| **Lf-L** (*n*=30) | 409.9 (140.2, 558.4) | | 253.3 (98.9, 596.8) | 0.465 | 257.5 (172.2, 342.9) | **Lf-L vs PL** | 32.1 (-24.5, 131.1) | 0.325 | |
| **PL** (*n*=34) | 333.5 (124.7, 520.0) | | 241.0 (107.1, 428.6) | 0.174 | 194.9 (107.5, 282.3) | **Lf-H vs Lf-L** | -0.4 (-37.1, 57.6) | 0.985 | |
| **IL-6** | |  |  |  |  |  |  |  |  |
| **Lf-H** (*n*=30) | 192.9 (113.3, 676.2) | | 227.6 (129.2, 421.7) | 0.719 | 259.4 (199.8, 319) | **Lf-H vs PL** | 34.5 (-11.3, 104.0) | 0.160 | |
| **Lf-L** (*n*=30) | 330.2 (212.0, 634.9) | | 185.4 (144.2, 409.9) | **0.009** | 200.9 (143.4, 258.3) | **Lf-L vs PL** | 4.2 (-33.1, 62.1) | 0.855 | |
| **PL** (*n*=33) | 302.5 (205.9, 558.4) | | 234.7 (123.1, 363.3) | **0.021** | 192.8 (130.7, 254.9) | **Lf-H vs Lf-L** | 29.2 (-11.6, 88.7) | 0.183 | |
| **TNF-α** | |  |  |  |  |  |  |  |  |
| **Lf-H** (*n*=30) | 64.9 (27.9, 114.6) | | 41.3. (26.9, 144.5) | 0.241 | 54.8 (40.4, 69.2) | **Lf-H vs PL** | 50.5 (0.2, 125.9) | **0.049** | |
| **Lf-L** (*n*=30) | 45.3 (24.7, 106.0) | | 37.4 (19.0, 63.4) | 0.086 | 33.6 (22.8, 44.4) | **Lf-L vs PL** | -7.8 (-41.9, 46.5) | 0.729 | |
| **PL** (*n*=33) | 50.2 (22.6, 83.4) | | 40.0 (17.0, 79.4) | 0.284 | 36.4 (25.2, 47.6) | **Lf-H vs Lf-L** | 63.2 (7.1, 148.5) | **0.023** | |
| PBMC, peripheral blood mononuclear cell. IQR, interquartile range.CI, confidence interval. IFN-γ, interferon gamma. IFN-α2, interferon alpha-2. IL-6, interleukin-6. TNF-α, tumour necrosis factor-alpha.  ^1^ No difference (*P*>0.05) in baseline values between intervention groups and placebo for all variables, analysed by Kruskal-Wallis Test. ^2^ Significant (*P*<0.05) within treatment group change analysed by Wilcoxon signed-rank test. ^3^Marginal means adjusted for baseline (pre) concentration, age, BMI, and time since vaccination. ^4^Model coefficients (odds ratios) of differences between groups were transformed and reported as percent [95% CI] difference in post variable between intervention groups, analysed by multiple linear regression model adjusted for baseline (pre) concentration, age, BMI and time since vaccination. | | | | | | | | | |

| **Table S8.** Circulating immune cell frequency in healthy, older adults at baseline (pre) and follow up (post) (unadjusted), and adjusted values at follow up (post), in 4-week intervention with high (Lf-H 600mg/d) or low dose (Lf-L, 200mg/d) oral lactoferrin or placebo (PL) | | | | | | | | | | | |
| --- | --- | --- | --- | --- | --- | --- | --- | --- | --- | --- | --- |
| **Outcome**  **Group** | **Pre**  Median (IQR) | | | | **Post**  Median (IQR) | ***P*^1^** | **Adjusted Post^2^**  Mean (95% CI) |  | **Difference in Post^3^**  % (95% CI) | | ***P*** |
| **Eosinophils** |  | | | |  |  |  |  | |  |  |
| **Lf-H** (*n*=30) | 21079.9 (13553.4, 33507.9) | | | | 20750.5 (17344, 28742.5) | 0.428 | 21667.1 (17860.6, 25473.7) | **Lf-H vs PL** | | 8.1 (-17.3, 41.3) | 0.565 |
| **Lf-L** (*n*=29) | 17600.4 (8986.6, 38453.2) | | | | 14539.5 (9553.8, 34960.3) | 0.738 | 18359.1 (13405.8, 23312.3) | **Lf-L vs PL** | | -8.4 (-34.7, 28.5) | 0.608 |
| **PL** (*n*=34) | 21263.2 (12703.2, 27860.2) | | | | 21634.9 (14674.1, 28021.2) | 0.352 | 20044.3 (16171.6, 23917.1) | **Lf-H vs Lf-L** | | 18 (-14.4, 62.8) | 0.309 |
| **Neutrophils** |  | | | |  |  |  |  | |  |  |
| **Lf-H** (*n*=29) | 921863.3 (895928.9, 947296.9) | | | | 531662.9 (489122, 616957.3) | 0.430 | 536502.3 (501675, 571329.6) | **Lf-H vs PL** | | -4.4 (-12, 4) | 0.291 |
| **Lf-L** (*n*=28) | 914906.4 (833869.3, 946924.4) | | | | 531417.6 (464713, 589736.3) | 0.210 | 509415.4 (470437.5, 548393.3) | **Lf-L vs PL** | | -9.2 (-17.3, -0.3) | **0.044** |
| **PL** (*n*=34) | 912074.9 (848282.1, 937439.7) | | | | 578649 (524116.9, 649262.3) | 0.089 | 560982.3 (531798, 590166.7) | **Lf-H vs Lf-L** | | 5.3 (-4.8, 16.5) | 0.312 |
| **T cells** |  | | | |  |  |  |  | |  |  |
| **Lf-H** (*n*=29) | 921863.3 (895928.9, 947296.9) | | | | 939979.6 (882590.1, 958047.9) | **0.033** | 915782.2 (875783.6, 955780.9) | **Lf-H vs PL** | | 7.5 (0.7, 14.8) | **0.031** |
| **Lf-L** (*n*=30) | 914906.4 (833869.3, 946924.4) | | | | 893515.8 (833951.6, 939228.2) | 0.441 | 860266.6 (829321, 891212.1) | **Lf-L vs PL** | | 1 (-5, 7.4) | 0.742 |
| **PL** (*n*=32) | 912074.9 (848282.1, 937439.7) | | | | 906368.5 (844568.3, 942341.8) | 0.290 | 851572.7 (811504.6, 891640.9) | **Lf-H vs Lf-L** | | 6.5 (0.4, 12.9) | **0.037** |
| **CD4^+^ T cells** |  | | | |  |  |  |  | |  |  |
| **Lf-H** (*n*=29) | 654420.9 (607461.2, 743569.5) | | | | 702415.3 (613604.6, 773590.1) | **0.013** | 683351.3 (638815.2, 727887.5) | **Lf-H vs PL** | | 9.9 (1.1, 19.5) | **0.028** |
| **Lf-L** (*n*=30) | 691798 (567441.7, 744921.4) | | | | 688550.3 (555846.4, 758436.6) | 0.349 | 634948.1 (606685.5, 663210.7) | **Lf-L vs PL** | | 2.1 (-4.3, 9) | 0.526 |
| **PL** (*n*=32) | 629819.8 (572882, 710791.6) | | | | 648030.1 (568536.9, 699281.4) | 0.597 | 621878.7 (592398.4, 651358.9) | **Lf-H vs Lf-L** | | 7.6 (-1, 17) | 0.084 |
| **Activated CD4^+^ cells** | | | |  |  |  |  |  | |  |  |
| **Lf-H** (*n*=29) | 44495.3 (28071.6, 74423.8) | | | | 48780.2 (40857.6, 69597) | 0.471 | 48367.6 (39895.4, 56839.8) | **Lf-H vs PL** | | -8.3 (-26, 13.6) | 0.422 |
| **Lf-L** (*n*=30) | 47453.3 (24705.5, 72273) | | | | 38260.3 (31252.1, 68432.5) | 0.229 | 43537.5 (33553.9, 53521.1) | **Lf-L vs PL** | | -17.5 (-37.4, 8.7) | 0.170 |
| **PL** (*n*=32) | 50837.5 (35547.6, 82134.3) | | | | 50382.4 (36325.4, 83197.1) | 0.164 | 52758.1 (46242.8, 59273.4) | **Lf-H vs Lf-L** | | 11.1 (-16.8, 48.4) | 0.471 |
| **CD8^+^ T cells** |  | | | |  |  |  |  | |  |  |
| **Lf-H** (*n*=29) | 215520 (154736.1, 286929.3) | | | | 204810.8 (149924.4, 262121.3) | 0.325 | 180900.7 (155386.4, 206414.9) | **Lf-H vs PL** | | 0.3 (-15, 18.4) | 0.974 |
| **Lf-L** (*n*=30) | 216511.3 (136488.3, 297040) | | | | 189255.5 (128621.1, 305955) | 0.299 | 161059 (124371.1, 197746.8) | **Lf-L vs PL** | | -10.7 (-30.5, 14.7) | 0.370 |
| **PL** (*n*=32) | 204729 (134631, 270690.1) | | | | 209240.8 (152948.3, 273602) | 0.057 | 180401.8 (165155.5, 195648.2) | **Lf-H vs Lf-L** | | 12.3 (-12.2, 43.7) | 0.351 |
| **Activated CD8^+^ T cells** | | | |  |  |  |  |  | |  |  |
| **Lf-H** (*n*=29) | 1417.5 (760.9, 3003.2) | | | | 903.7 (707.5, 2936.6) | 0.848 | 1646.7 (1070.5, 2222.9) | **Lf-H vs PL** | | -18.7 (-48.1, 27.3) | 0.361 |
| **Lf-L** (*n*=29) | 1732.6 (835.5, 3195.7) | | | | 1297.9 (515.5, 2092.1) | 0.112 | 1186.5 (726.4, 1646.6) | **Lf-L vs PL** | | -41.4 (-64, -4.8) | **0.031** |
| **PL** (*n*=32) | 2018.5 (996.6, 4031) | | | | 2194.6 (885.5, 3815.3) | 0.875 | 2025.5 (1506, 2545) | **Lf-H vs Lf-L** | | 38.8 (-17.4, 133.3) | 0.213 |
| **Regulatory T cells** | | |  | |  |  |  |  | |  |  |
| **Lf-H** (*n*=29) | 43243.5 (34779.6, 54093.9) | | | | 44264.2 (39113.6, 49510.3) | 0.149 | 46115.2 (42289.6, 49940.8) | **Lf-H vs PL** | | 4.8 (-6.4, 17.4) | 0.409 |
| **Lf-L** (*n*=30) | 42653.6 (31962.5, 54207.6) | | | | 44280.5 (32179.3, 57179.8) | 0.441 | 42170.9 (36421.5, 47920.3) | **Lf-L vs PL** | | -4.1 (-18.5, 12.7) | 0.606 |
| **PL** (*n*=32) | 45763.1 (33579.3, 58775.4) | | | | 45552.8 (35416.3, 57236.2) | 0.367 | 43984.1 (40694.5, 47273.6) | **Lf-H vs Lf-L** | | 9.4 (-6.7, 28.1) | 0.264 |
| **γδ T cells** |  | | | |  |  |  |  | |  |  |
| **Lf-H** (*n*=29) | 19475.2 (12128.4, 35843.9) | | | | 17580.6 (11226.8, 35907.6) | 0.428 | 17499.5 (11966, 23032.9) | **Lf-H vs PL** | | -32.1 (-53.5, -0.8) | **0.046** |
| **Lf-L** (*n*=30) | 32960.4 (19739.4, 49621.6) | | | | 27850.7 (18176.9, 49917) | 0.975 | 18423.9 (14183.1, 22664.8) | **Lf-L vs PL** | | -28.5 (-47.2, -3.1) | **0.031** |
| **PL** (*n*=32) | 17835.6 (7979, 36728.4) | | | | 18133.7 (9594.6, 40858.3) | 0.126 | 25760.6 (22010.4, 29510.9) | **Lf-H vs Lf-L** | | -5 (-33.5, 35.6) | 0.774 |
| **BDCA-1 DCs** | |  | | |  |  |  |  | |  |  |
| **Lf-H** (*n*=30) | 113636.4 (73792.7, 172946.5) | | | | 118164.2 (89128.7, 165105.4) | 0.428 | 118538 (92433.5, 144642.5) | **Lf-H vs PL** | | 24.9 (-5, 64.3) | 0.110 |
| **Lf-L** (*n*=30) | 119119.9 (79181.5, 142631.6) | | | | 88017 (55819.5, 126984.1) | 0.060 | 80300.4 (62712.6, 97888.2) | **Lf-L vs PL** | | -15.4 (-35.9, 11.8) | 0.237 |
| **PL** (*n*=34) | 110155.8 (67362.1, 150000) | | | | 91075.4 (62141.5, 140671.3) | 0.209 | 94878.2 (79529.4, 110227.1) | **Lf-H vs Lf-L** | | 47.6 (7.8, 102.1) | **0.016** |
| **BDCA-3 DCs** | |  | | |  |  |  |  | |  |  |
| **Lf-H** (*n*=29) | 100377.2 (53493.7, 173594.1) | | | | 93913 (45791.7, 154676.3) | 0.294 | 84102.3 (34558.5, 133646) | **Lf-H vs PL** | | -41.4 (-70.5, 16.5) | 0.126 |
| **Lf-L** (*n*=30) | 81942.3 (36772.3, 193979.9) | | | | 67771.3 (27777.8, 150697.7) | 0.504 | 80040.2 (40054.3, 120026.2) | **Lf-L vs PL** | | -44.2 (-72.9, 14.7) | 0.111 |
| **PL** (*n*=34) | 94432.2 (65934.1, 169105.7) | | | | 115809.6 (61643.8, 191016.8) | 0.626 | 143530.6 (61409, 225652.1) | **Lf-H vs Lf-L** | | 5.1 (-47.9, 111.9) | 0.889 |
| **pDCs** |  | | | |  |  |  |  | |  |  |
| **Lf-H** (*n*=29) | 46074.7 (28154.6, 56701) | | | | 45422.8 (35971.2, 58608.1) | 0.552 | 42612.8 (33961, 51264.7) | **Lf-H vs PL** | | 24.2 (-8.6, 68.6) | 0.163 |
| **Lf-L** (*n*=30) | 43718.6 (30375.7, 60344.8) | | | | 38139.1 (17985.6, 64426.7) | 0.339 | 33689.9 (24829.9, 42549.8) | **Lf-L vs PL** | | -1.8 (-30, 37.6) | 0.913 |
| **PL** (*n*=34) | 30055.6 (22341.4, 57894.7) | | | | 33949.5 (26017.8, 43750) | 0.651 | 34320.8 (27221.6, 41420) | **Lf-H vs Lf-L** | | 26.5 (-9.2, 76.2) | 0.163 |
| **B cells** |  | | | |  |  |  |  | |  |  |
| **Lf-H** (*n*=29) | 71908.5 (38922, 178220.5) | | | | 113163.4 (36870, 150585.3) | 0.538 | 72054.4 (54076.3, 90032.5) | **Lf-H vs PL** | | -18.2 (-40.1, 11.9) | 0.206 |
| **Lf-L** (*n*=30) | 73516 (45253.3, 110293.3) | | | | 79487.4 (43877.6, 121524.4) | 0.797 | 68763.6 (48997.2, 88530) | **Lf-L vs PL** | | -21.9 (-44.7, 10.3) | 0.159 |
| **PL** (*n*=34) | 85502.7 (51109.1, 143908.7) | | | | 84917.1 (60868, 138564.8) | 0.818 | 88043.8 (72088.4, 103999.1) | **Lf-H vs Lf-L** | | 4.8 (-28.3, 53.1) | 0.807 |
| **NK cells** |  | | | |  |  |  |  | |  |  |
| **Lf-H** (*n*=29) | 23984.3 (8693, 36926.8)^4^ | | | | 25234.1 (5561.3, 66523.8) | 0.347 | 16843.6 (7233.1, 26454.2) | **Lf-H vs PL** | | -19.3 (-57.8, 54.3) | 0.512 |
| **Lf-L** (*n*=27) | 22941 (6950.5, 55226.1)^4^ | | | | 17126.2 (7192.1, 44891) | 0.080 | 11952.1 (6434.6, 17469.5) | **Lf-L vs PL** | | -42.7 (-66.8, -1.2) | **0.045** |
| **PL** (*n*=34) | 46294.9 (24081.7, 80359.5) | | | | 39238.8 (18788.1, 64185) | 0.242 | 20873 (12421.2, 29324.8) | **Lf-H vs Lf-L** | | 40.9 (-25, 164.9) | 0.283 |
| IQR, interquartile range. CI, confidence interval. BDCA, blood dendritic cell antigen. DC, dendritic cell. NK, natural killer.  ^1^ Within treatment group change analysed by Wilcoxon signed-rank test. ^2^ Marginal means adjusted for baseline (pre) concentration, age, BMI, and time since vaccination. ^3^Model coefficients (odds ratios) of differences between groups were transformed and reported as percent [95% CI] difference in post variable between intervention groups, analysed by multiple linear regression model adjusted for baseline (pre) concentration, age, BMI and time since vaccination. ^4^Significant difference (*P*<0.05) in baseline values between intervention group and placebo, analysed by Kruskal-Wallis Test. | | | | | | | | | | | |

| **Table S9.** Systemic Inflammation in healthy, older adults at baseline (pre) and follow up (post) (unadjusted), and adjusted values at follow up (post), in 4-week intervention with high (Lf-H 600mg/d) or low dose (Lf-L, 200mg/d) oral lactoferrin or placebo (PL) | | | | | | | | | | | | | | | | | | | |  |  |
| --- | --- | --- | --- | --- | --- | --- | --- | --- | --- | --- | --- | --- | --- | --- | --- | --- | --- | --- | --- | --- | --- |
| **Outcome**  **Group** | **Pre^1^**  Median (IQR) | | | **Post**  Median (IQR) | | | ***P*^2^** | **Adjusted Post**^3^  Mean (95% CI) | | | |  | **Difference in Post^4^**  % (95% CI) | | | | | ***P*** | |  |  |
| **IL-6** | |  |  | | | |  |  | | |  | | | |  |  | | | | |  |
| **Lf-H** (*n*=31) | | 1.2 (0.7, 1.5) | | | 0.9 (0.7, 1.4) | **0.046** | | | 1.0 (0.8, 1.1) | **Lf-H vs PL** | | | | -16.3 (-32.3, 3.5) | | | 0.100 | |  |  |  |
| **Lf-L** (*n*=30) | | 1.0 (0.7, 1.4) | | | 1.1 (0.8, 1.7) | 0.088 | | | 1.3 (1.1, 1.5) | **Lf-L vs PL** | | | | 13.2 (-9.1, 40.9) | | | 0.266 | |  |  |  |
| **PL** (*n*=34) | | 1.0 (0.7, 1.6) | | | 1.1 (0.7, 1.6) | 0.798 | | | 1.2 (1.0, 1.3) | **Lf-H vs Lf-L** | | | | -26.0 (-39.5, -9.5) | | | **0.004** | |  |  |  |
| **CRP** | |  | | |  |  | | |  |  | | | |  | | |  | |  |  |  |
| **Lf-H** (*n*=31) | | 1.3 (0.6, 2.1) | | | 0.9 (0.6, 2.0) | 0.070 | | | 1.1 (0.8, 1.3) | **Lf-H vs PL** | | | | -11.1 (-36.2, 23.9) | | | 0.483 | |  |  |  |
| **Lf-L** (*n*=30) | | 1.3 (0.5, 2.4) | | | 1.4 (0.8, 3.2) | 0.422 | | | 1.5 (1.2, 1.9) | **Lf-L vs PL** | | | | 28.1 (-9.1, 80.5) | | | 0.155 | |  |  |  |
| **PL** (*n*=34) | | 1.1 (0.6, 2.3) | | | 1.1 (0.6, 2.7) | 0.858 | | | 1.2 (0.9, 1.5) | **Lf-H vs Lf-L** | | | | -30.6 (-49.7, -4.4) | | | **0.026** | |  |  |  |
| **TNF-α** | |  | | |  |  | | |  |  | | | |  | | |  | |  |  |  |
| **Lf-H** (*n*=31) | | 0.8 (0.6, 1.0) | | | 0.8 (0.6, 0.9) | 0.411 | | | 0.8 (0.8, 0.9) | **Lf-H vs PL** | | | | 1.8 (-8.6, 13.4) | | | 0.741 | |  |  |  |
| **Lf-L** (*n*=30) | | 0.8 (0.7, 1.0) | | | 0.8 (0.6, 1.0) | 0.405 | | | 0.9 (0.8, 1.0) | **Lf-L vs PL** | | | | 11 (-3.6, 27.8) | | | 0.144 | |  |  |  |
| **PL** (*n*=34) | | 0.9 (0.6, 1.0) | | | 0.8 (0.7, 1.0) | 0.885 | | | 0.8 (0.7, 0.9) | **Lf-H vs Lf-L** | | | | -8.3 (-18.2, 2.8) | | | 0.137 | |  |  |  |
| IQR, interquartile range. IL-6, interleukin-6. CRP, C-reactive protein. TNF- α, tumour necrosis factor-alpha.  ^1^ No difference (*P*>0.05) in baseline values between intervention groups and placebo for all variables, analysed by Kruskal-Wallis Test. ^2^ Significant (*P*<0.05) within treatment group change analysed by Wilcoxon signed-rank test. ^3^Marginal means adjusted for baseline (pre) concentration, age, BMI, and time since vaccination. ^4^Model coefficients (odds ratios) of differences between groups were transformed and reported as percent [95% CI] difference in post variable between intervention groups, analysed by multiple linear regression model adjusted for baseline (pre) concentration, age, BMI and time since vaccination. | | | | | | | | | | | | | | | | | | | | | |

| **Table S10 A.** Health-Related Quality of Life (HRQOL-14) in healthy, older adults at baseline (pre) and follow up (post), in 4-week intervention with high (Lf-H 600mg/d) or low dose (Lf-L, 200mg/d) oral lactoferrin or placebo (PL). | | | | | | | | | | |
| --- | --- | --- | --- | --- | --- | --- | --- | --- | --- | --- |
| **Outcome** | **Pre**^1^ | | | | **Post** | **Difference Between Groups**^3^ | | | | |
| **Group** | | Median (IQR) or Mean±SD^2^ | | | |  | β (95% CI) | | | ***P*** |
| **Healthy Days Module** | | | | | | | | | | |
| **General Health Rating**^4,5^ | | | | | | | | | | |
| **Lf-H** (*n*=31) | | | 2.0 ± 0.7 | 2.1 ± 0.8 | | **Lf-H vs PL** | | 0.1 (-0.2, 0.4) | 0.489 | |
| **Lf-L** (*n*=31) | | | 1.9 ± 0.7 | 1.9 ± 0.7 | | **Lf-L vs PL** | | -0.1 (-0.3, 0.2) | 0.693 | |
| **PL** (*n*=34) | | | 2.3 ± 0.8 | 2.3 ± 0.7 | | **Lf-H vs Lf-L** | | 0.2 (-0.1, 0.5) | 0.286 | |
| **Healthy days**^6,7^ | | | | | | | | | | |
| **Lf-H** (*n*=31) | | | 29.0 (24.0, 30.0) | 27.0 (22.0, 30.0) | | **Lf-H vs PL** | | 0.0 (-0.2, 0.1) | 0.918 | |
| **Lf-L** (*n*=31) | | | 30.0 (26.0, 30.0) | 30.0 (22.0, 30.0) | | **Lf-L vs PL** | | 0.0 (-0.1, 0.2) | 0.849 | |
| **PL**  (*n*=34) | | | 28.0 (25.0, 30.0) | 26.5 (23.0, 29.0) | | **Lf-H vs Lf-L** | | 0.0 (-0.2, 0.2) | 0.932 | |
| **Unhealthy days**^6,8^ | | | | | | | | | | |
| **Lf-H** (*n*=31) | | | 1.0 (0.0, 6.0) | 3.0 (0.0, 8.0) | | **Lf-H vs PL** | | -0.1 (-1.1, 0.8) | 0.776 | |
| **Lf-L** (*n*=31) | | | 0.0 (0.0, 4.0) | 0 .0(0.0, 8.0) | | **Lf-L vs PL** | | -0.2 (-1.1, 0.8) | 0.749 | |
| **PL** (*n*=34) | | | 2.0 (0.0, 5.0) | 3.5 (1.0, 7.0) | | **Lf-H vs Lf-L** | | 0.0 (-0.9, 1.0) | 0.965 | |
| **Health impact on activity days**^9,10^ | | | | | | | | | | |
| **Lf-H** (*n*=18) | | | 0.0 (0.0, 1.0) | 0.0 (0.0, 4.0) | | **Lf-H vs PL** | | 1.2 (0.4, 2.0) | **0.003** | |
| **Lf-L** (*n*=16) | | | 0.0 (0.0, 1.5) | 0.0 (0.0, 5.0) | | **Lf-L vs PL** | | 0.0 (-0.8, 0.9) | 0.918 | |
| **PL** (*n*=21) | | | 0.0 (0.0, 2.0) | 0.0 (0.0, 2.0) | | **Lf-H vs Lf-L** | | 1.1 (0.4, 1.9) | **0.003** | |
| IQR; interquartile range.CI, confidence interval.^1^No difference (p>0.05) in baseline values between intervention groups for all variables analysed by linear mixed-effects model. ^2^Skewed data are presented as median (IQR), normally distributed data are presented as mean±SD. ^3^Coefficient [95% CI] of difference in variable change over time between intervention groups. ^4^Analysed by Linear mixed-effects model. ^5^Question scores are Excellent=1, Very good =2, Good=3, Fair=4, Poor=5. ^6^Analysed by mixed-effects Negative Binomial model. ^7^Number of recent days when a person's physical and mental health was good (or better). ^8^Number of recent days when a person’s physical or mental health was not good. ^9^Analysed by Mixed-effects Poisson regression model. ^10^Number of days poor physical or mental health interfered with usual activities – response recorded when ‘Healthy Days’ <30. | | | | | | | | | | |

| **Table S10 B.**  Health-Related Quality of Life (HRQOL-14) in healthy, older adults at baseline (pre) and follow up (post), in 4-week intervention with high (Lf-H 600mg/d) or low dose (Lf-L, 200mg/d) oral lactoferrin or placebo (PL). | | | | | | | | | |
| --- | --- | --- | --- | --- | --- | --- | --- | --- | --- |
| **Outcome** | **Pre**^1^ | | | | **Post** | **Difference Between Groups**^3^ | | | |
| **Group** | | Median (IQR) or Mean±SD^2^ | | | |  | β (95% CI) | | ***P*** |
| **Healthy Days Symptoms Module** | | | | | | | | | |
| **Pain days**^4^ | | | | | | | | | |
| **Lf-H** (*n*=31) | | | 0.0 (0.0, 0.0) | 0.0 (0.0, 2.0) | | **Lf-H vs PL** | 1.3 (-0.3, 3.0) | 0.120 | |
| **Lf-L** (*n*=31) | | | 0.0 (0.0, 0.0) | 0.0 (0.0, 0.0) | | **Lf-L vs PL** | 0.5 (-1.3, 2.2) | 0.599 | |
| **PL** (*n*=34) | | | 0.0 (0.0, 2.0) | 0.0 (0.0, 0.0) | | **Lf-H vs Lf-L** | 0.9 (-0.9, 2.6) | 0.332 | |
| **Sad days**^4^ | | | | | | | | | |
| **Lf-H** (*n*=31) | | | 0.0 (0.0, 3.0) | 0.0 (0.0, 3.0) | | **Lf-H vs PL** | -0.1 (-1.3, 1.1) | 0.819 | |
| **Lf-L** (*n*=31) | | | 0.0 (0.0, 2.0) | 0.0 (0.0, 0.0) | | **Lf-L vs PL** | -1.0 (-2.3, 0.2) | 0.114 | |
| **PL** (*n*=34) | | | 0.0 (0.0, 3.0) | 1.0 (0.0, 4.0) | | **Lf-H vs Lf-L** | 0.9 (-0.4, 2.2) | 0.191 | |
| **Anxious days**^4^ | | | | | | | | | |
| **Lf-H** (*n*=31) | | | 0.0 (0.0, 5.0) ^a^ | 2.0 (0.0, 5.0) | | **Lf-H vs PL** | 0.2 (-0.9, 1.3) | 0.711 | |
| **Lf-L** (*n*=31) | | | 0.0 (0.0, 2.0) ^b^ | 0.0 (0.0, 3.0) | | **Lf-L vs PL** | 0.4 (-0.7, 1.6) | 0.481 | |
| **PL** (*n*=34) | | | 1.0 (0.0, 3.0) ^a,c^ | 2.0 (0.0, 5.0) | | **Lf-H vs Lf-L** | -0.2 (-1.4, 1.0) | 0.724 | |
| **Sleep problem days**^4^ | | | | | | | | | |
| **Lf-H** (*n*=31) | | | 3.0 (0.0, 7.0) | 3.0 (0.0, 6.0) | | **Lf-H vs PL** | 0.1 (-0.7, 0.8) | 0.882 | |
| **Lf-L** (*n*=31) | | | 2.0 (0.0, 7.0) | 2.0 (0.0, 7.0) | | **Lf-L vs PL** | 0.2 (-0.5, 1.0) | 0.547 | |
| **PL** (*n*=34) | | | 2.0 (0.0, 5.0) | 1.0 (0.0, 6.0) | | **Lf-H vs Lf-L** | -0.2 (-0.9, 0.6) | 0.643 | |
| **Energetic days**^5^ | | | | | | | | | |
| **Lf-H** (*n*=31) | | | 26.0 (15.0, 30.0) | 25.0 (15.0, 28) | | **Lf-H vs PL** | -0.2 (-0.3, -0.0) | **0.045** | |
| **Lf-L** (*n*=31) | | | 26.0 (15.0, 30.0) | 27.0 (20.0, 30.0) | | **Lf-L vs PL** | 0.0 (-0.2, 0.1) | 0.765 | |
| **PL** (*n*=34) | | | 25.0 (10.0, 28.0) | 24.5 (17.0, 30.0) | | **Lf-H vs Lf-L** | -0.1 (-0.3, 0.0) | 0.089 | |
| IQR; interquartile range.CI, confidence interval. ^1^No difference (p>0.05) in baseline values between intervention groups for all variables analysed by linear mixed-effects model. ^2^Skewed data are presented as median (IQR), normally distributed data are presented as mean±SD. ^3^Coefficient [95% CI] of difference in variable change over time between intervention groups. ^4^Analysed by mixed-effects Negative Binomial model. ^5^Analysed by Mixed-effects Poisson regression model. | | | | | | | | | |

**Figure S1. Flow cytometry gating strategy used to identify immune cell subsets**

Whole blood immune cells were labelled and quantified in 4 separate tubes: **A**: Granulocyte subsets, **B**: γδ, αβ, CD4, CD8, and Treg cell subsets, **C**: DC subsets, **D**: B cells and NK cell subsets. Akt, activated. CD, cluster of differentiation. DC, dendritic cells. NK, natural killer. TCR, T cell receptor. Treg, T regulatory.

**
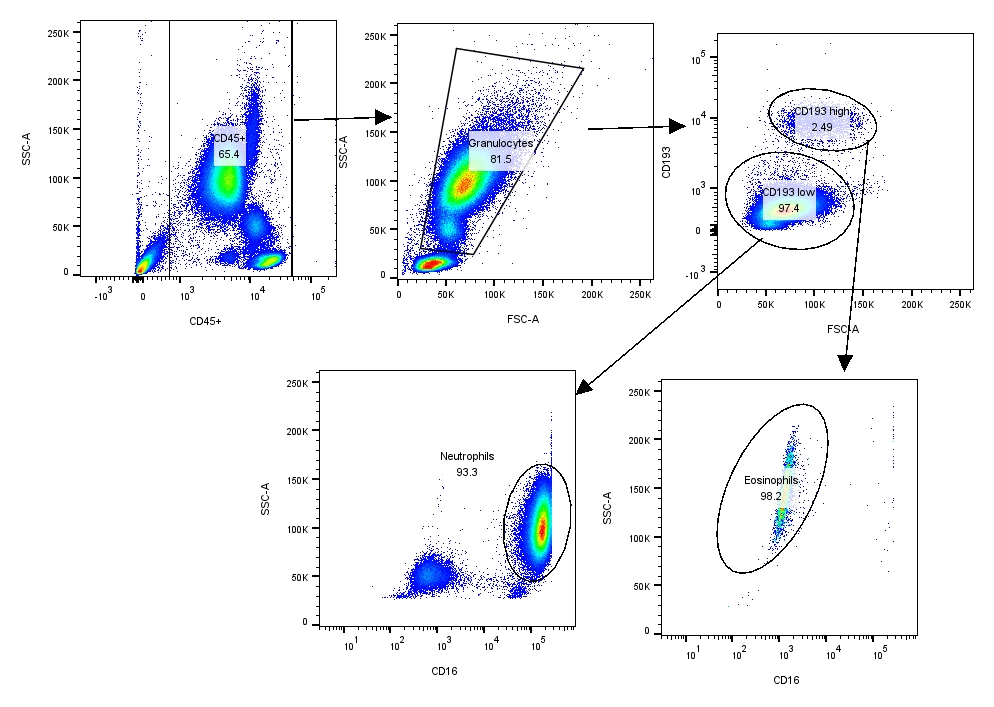
Figure S1 A**

**
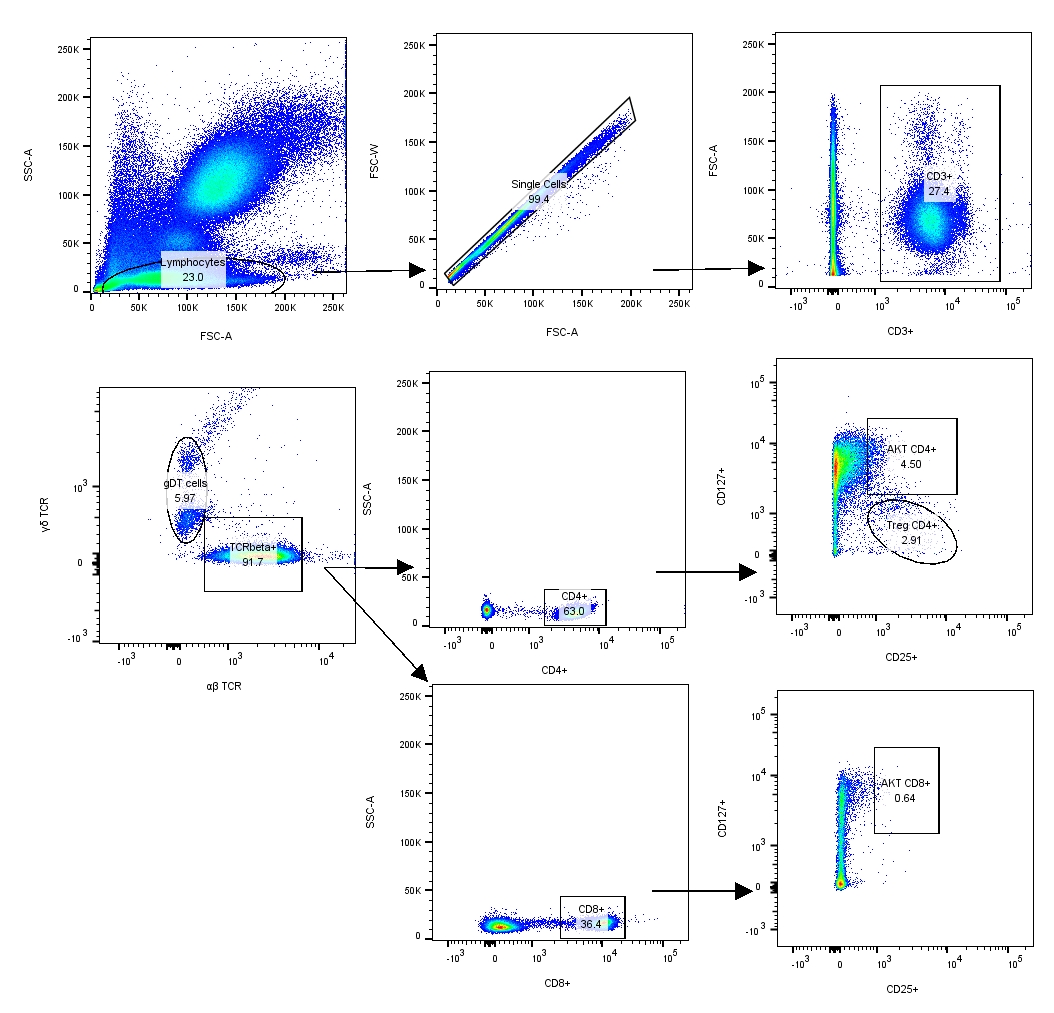
Figure S1 B**


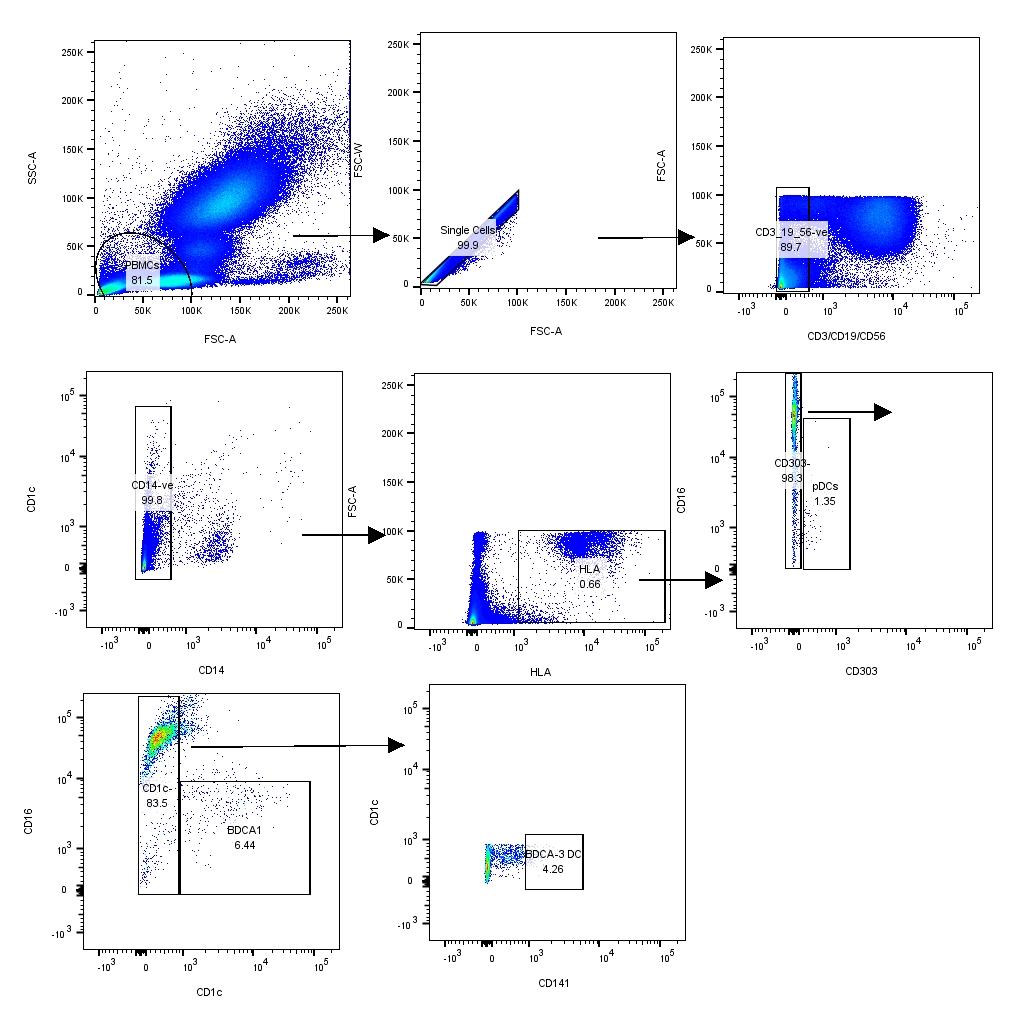
**Figure S1 C**


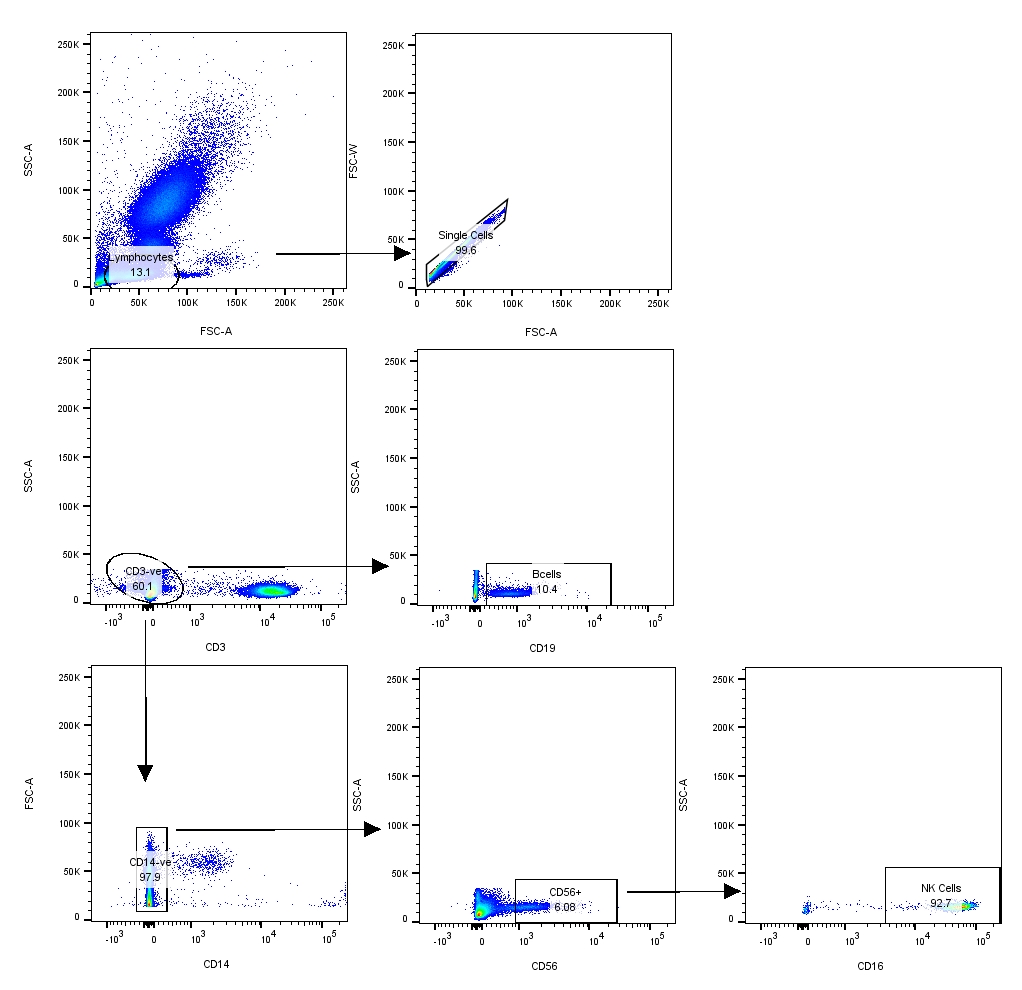
**Figure S1 D**

**
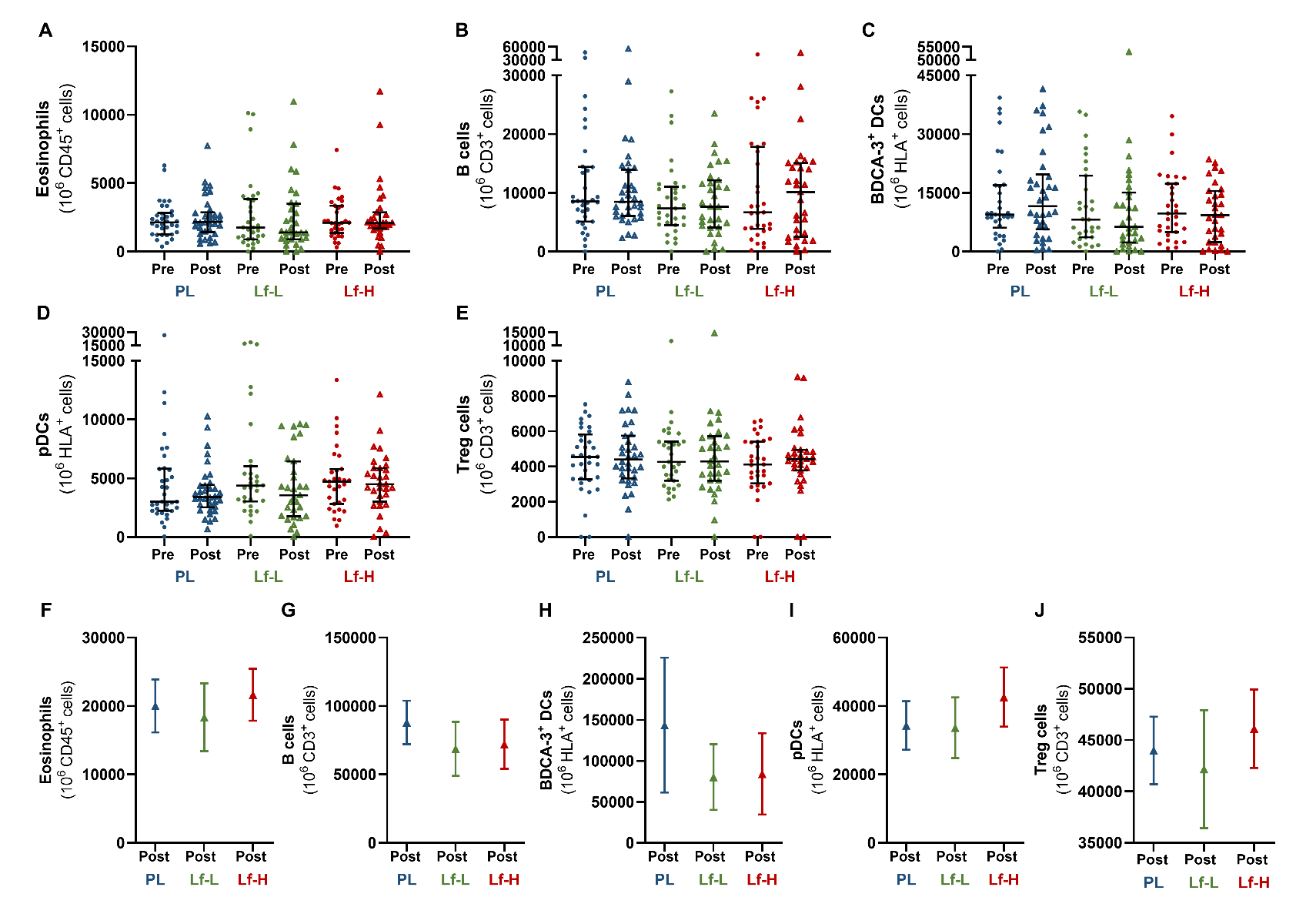
Figure S2. Circulating immune cell frequency in peripheral blood** **from healthy, older adults at baseline (pre) and follow up (post) (unadjusted A-E) and adjusted (F-J) frequency at follow up (post), in 4-week intervention with high dose (Lf-H) or low dose (Lf-L) oral lactoferrin or placebo (PL). A, F:** Eosinophils. **B, G:** B Cells. **C, H:** BDCA-3^+^ DCs. **D, I:** pDCs. **E, J**: T reg cells. A-E: pre and post data are displayed as unadjusted medians with interquartile range; F-J: post data displayed as marginal means (95% CI) adjusted for baseline (pre) concentration, age, BMI and time since vaccination. No differences (P>0.05) at baseline between intervention groups and placebo, analysed by Kruskal-Wallis Test. Within treatment group change analysed by Wilcoxon signed-rank test. Difference in post between intervention groups analysed by multiple linear regression model adjusted for baseline (pre) concentration, age, BMI and time since vaccination. **P*<0.05. BDCA, blood dendritic cell antigen. DC, dendritic cell. T reg, regulatory T cells.

**Supplemental References**

1. NIH Clinical Center Healthy Volunteers. <https://www.cc.nih.gov/recruit/volunteers> (acessed January 2025
