# Supplementary material for: Oral lactoferrin reduces systemic inflammation, enhances anti-viral responses and modulates immune cell profiles: a randomised controlled trial in healthy, older adults
Source: Br J Nutr. 2026 Feb 4;135(9):929–43. doi: 10.1017/S000711452610631X (PMC13315551; doi:10.1017/S000711452610631X)
Supplement: Berthon et al. supplementary material 2 — Berthon et al. supplementary material [file S000711452610631Xsup002.pdf]

Lactoferrin Supplementation, Immune Function & Respiratory Virus Infection:

The "LIFE" Clinical Trial

Research Protocol

HNE HREC / NSW REGIS ETHICS Reference: 2021/ETH10928
UoN HREC Reference No: H-2021-0334
ANZCTR Reference No: ACTRN12621001511820
Universal Trial Number: U1111-1268-3042
TGA CTN Reference No: CT-2021-CTN-04015-1-v1

Chief Investigator
Professor Lisa Wood
Nutrition and Immunometabolism, Immune Health Research Program, Hunter Medical Research Institute, Lot 1 Kookaburra Circuit, New Lambton Heights, NSW, 2305
School of Biomedical Science and Pharmacy, University of Newcastle, Newcastle, NSW

Co-Investigators
Associate Professor Nathan Bartlett
Doctor Bronwyn Berthon
Doctor Evan Williams
Doctor Lily Williams

Sponsor:
This trial is funded by a Commonwealth of Australia, Department of Industry, Innovation and Science Innovations Connection Project Grant in partnership with Noumi Limited and the University of Newcastle.

PROTOCOL VERSION CONTROL

Version	Date	Prepared By	Change Description	Approved By
1	06.07.2021	B Berthon	N/A	L Wood
2	07.09.2021	B Berthon	Changes in response to ethical review	L Wood
3	01/02/2022	B.Berthon	Additional recruitment strategies	L. Wood
4	28/04/2022	B.Berthon	Change to participant instructions & eligibility criteria	L. Wood
5	22/08/2025	B.Berthon	Formatted for publication	L. Wood

This work is licensed under a Creative Commons Attribution 4.0 International License.

Contents

1. Purpose of Trial **3**

2. Aims **3**

3. Study design **3**

3.1 Intervention 4

3.2 Inclusion Criteria 4

3.3 Exclusion criteria 4

4. Methods and Procedures **4**

4.1.1 Participant recruitment 4

4.1.2 Trial visits 5

4.1.3 Outcome measures 7

4.1.4 Measurement techniques 7

4.1.5 Adherence 9

4.2 Statistical Analysis Plan 9

4.3 Sample size 10

4.4 Randomisation 10

5. Investigational product **10**

5.1.1 Description 10

5.1.2 Safety 11

5.1.3 Digestion, absorption and excretion 11

5.1.4 Manufacturer 11

5.1.5 IP Summary 12

6. Safety and Ethical Considerations **12**

6.1 Safety 12

6.1.1 Reporting of Adverse Events 13

6.1.2 Quality Control and Quality Assurance 14

6.1.3 Discontinuation 14

6.1.4 Disclosure, Publication and Confidentiality 14

6.1.5 Informed Consent, Ethical Review, and Regulatory Considerations 14

7. References **15**

1. Purpose of Trial

Bovine lactoferrin is a protein found in cow's milk which has been shown to modulate the immune system and reduce systemic inflammation. Lactoferrin supplementation has been shown to be safe, and without adverse effects in studies using up to 1000mg of Lf per day. the literature shows dosages of both 200mg and 600mg have been associated with improvements in systemic inflammation and immune function. Thus, this trial will serve as a dose finding trial, testing 0, 200mg and 600mg daily dose, to determine the minimal effective dose. This trial will investigate the effects of bovine lactoferrin supplementation on immune function and systemic inflammation in healthy, older adults. Immune function declines with increasing age, with a dampening of both adaptive and innate immune responses shown in older adults [1]. This intervention is proposed to improve immune function, thus older adults are a population who may be most likely to gain benefit from the intervention.

2. Aims

In healthy, older adults, we aim to examine the effect of both high dose and low dose bovine lactoferrin supplementation on:

1. Immune cell responses to respiratory virus infection,

2. Peripheral immune cell profiles, and

3. Systemic inflammation.

3. Study design

A double-blind, randomised, placebo-controlled, 4-week intervention trial, with 3 arms in parallel, including healthy, older adults. The trial will assess the efficacy of both high dose and low dose bovine lactoferrin supplementation in improving immune function, by measuring cytokine release from virus-infected peripheral blood mononuclear cells (PBMCs). The effect of bovine lactoferrin on peripheral immune cell profiles and systemic inflammation will also be examined.

The research will be conducted in accordance with ICH GCP standards, approved by Hunter New England Human Research Ethics Committee, and registered with University of Newcastle Human Research Ethics Committee and the Australian New Zealand Clinical Trials Registry (ANZCTR).

3.1 Intervention

All participants will be randomised at the baseline visit, to receive a 4-week intervention with either:

1. High dose lactoferrin: 600mg/d (2 x 300mg capsules), or
2. Low dose lactoferrin: 200mg (2 x 100mg capsules), or
3. Placebo control: 2 x placebo (Microcrystalline cellulose) capsules.

Participants will be instructed to consume 2 capsules per day with water, in the morning, 30 minutes before food.

3.2 Inclusion Criteria

- Males and females aged ≥50 years of age
- Non-smokers (ceased smoking ≥6 months prior)

3.3 Exclusion criteria

- Cow's milk allergy (self-reported)
- Use of any product (e.g. dietary or nutritional supplements) containing lactoferrin.
- Irregular use of dietary or nutritional supplements. If appropriate to do so, supplements being used inconsistently or containing lactoferrin, that are not being taken for a health condition may be washed out for 2-4 weeks before commencing the trial
- Current smokers (smoked within previous 6 months)
- Maintenance use of systemic corticosteroid, immunosuppressive or antibiotic drugs
- Unstable cardiac, renal, hypertensive, pulmonary, endocrine, immunologic, neurologic disorders
- Acute or terminal illness, human immunodeficiency virus (HIV) or active cancer
- Any other medical condition which may interfere with the participant's ability to participate in the intervention.

4. Methods and Procedures

4.1.1 Participant recruitment

The recruitment strategy for this trial will include inviting people from the existing Department of Respiratory and Sleep Medicine Outpatient database and the HMRI research volunteer register, which include participants that have expressed interest in participating in research and have consented to be contacted for future research projects. Other recruitment strategies will include advertising within the community using the HMRI newsletter, flyers, a media release, social media platforms and shopa docket ads at local chemists.

The recruitment strategy uses multiple avenues that allow potential participants to actively approach the research team to participate. The research team will only contact people who have already consented to be contacted for research within our research databases, or those who have contacted the team from advertising in social or traditional media.

Initially, interested potential participants will be contacted by telephone and provided with the participant information and consent form, either by post or email, and given time to consider whether they would like to participate and discuss the project with others such as their general practitioner. A contact number will be given to the potential participant, should they wish to discuss any aspect of the trial. After 1-2 weeks the trial coordinator will phone the potential participant to determine whether they would like to participate and answer any questions they may have. If they are interested, a telephone screen will then take place. The telephone screen is conducted to determine eligibility of potential participants. Participants will firstly be asked if they are willing to take part in the trial and if they are, preliminary assessment of their eligibility will be conducted based on the inclusion and exclusion criteria of the trial. Then, if appropriate, a mutually convenient time will be arranged to attend the baseline trial visit.

At the baseline trial visit participants will have the opportunity again to ask any questions about the project before informed consent is obtained.

Baseline visits will be postponed for at least 4 weeks for any participant who has required antibiotics or oral corticosteroids within the previous month, or if they are experiencing a current acute illness; and postponed for at least 4 weeks following vaccination (including seasonal influenza vaccines and COVID-19 vaccines). In addition, participants will be asked to avoid scheduling vaccination appointments during the trial 4-week intervention period. Participants will not be asked to delay vaccination appointments; trial visits will be scheduled accordingly.

4.1.2 Trial visits

Participants will be screened for trial eligibility by telephone prior to trial commencement. Eligible participants will be randomised and booked to attend the HMRI clinic for the baseline (V1) and final visit (V2) at mutually convenient times. Participants will be phoned prior to their appointments to confirm that they are able to attend. During the intervention, participants will also be telephoned once, after 2 weeks (**Figure 1, Table 1).**

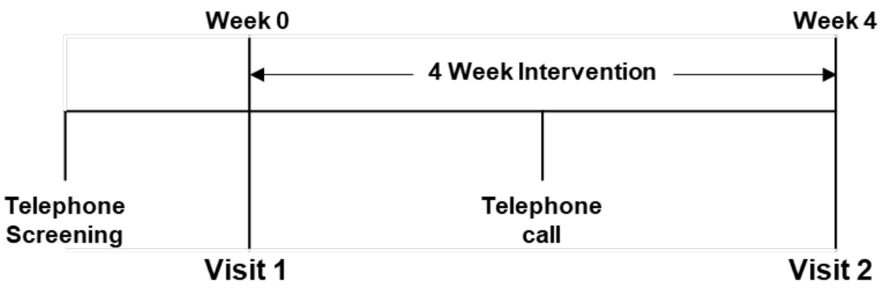

Figure 1. Trial visit schedule

Telephone screening: Interested participants will be screened for trial eligibility by telephone, including smoking status, food allergies and intolerances, brief medical history, and medication usage. Eligible participants will be invited to attend the HMRI clinic for visit 1.

Baseline Visit (V1): Participants will attend the Clinical Trials Facility on Level 4 at the Hunter Medical Research Institute (HMRI), after a 12-hour overnight fast. At this baseline visit, participants will have the opportunity to ask and have their questions satisfactorily answered by the trial coordinator before signing the consent form. Medical history and medication use will be recorded. Participants will also be asked to complete a food frequency questionnaire to assess usual dietary intake, and a 24-hour food recall with frequency checklist will be collected to assess recent dietary intake. Participants will also complete a health-related quality of life questionnaire (HRQOL-14). Height, body weight and blood pressure will be measured. A venous blood sample (36ml) will be collected via venepuncture by trained personnel. Blood samples will be processed and stored on site (level 2, West Wing, HMRI).

At the completion of the visit participants will receive their trial supplement, to be taken once daily, with water before food in the morning, for 4 weeks. For the duration of the trial, participants will complete a trial diary entry each day to record compliance with the trial supplement, any illnesses, and adverse effects.

Telephone follow-up (PC1): Participants will be contacted by telephone at week 2 for motivational purposes, to collect information on compliance with the trial supplement and to establish whether any adverse events or illness have been experienced.

Final Visit (V2): Participants will be phoned the day prior to their appointment to confirm they are able to attend and will be asked to bring to their appointment; their trial diary, and any remaining capsules of the trial supplement. Four weeks (28 days) after the baseline visit, participants will arrive at the HMRI clinic after a 12-hour overnight fast. Body weight and blood pressure will be measured. Participants will complete a health-related quality of life

questionnaire (HRQOL-14), and a 24-hour food recall with frequency checklist will be collected to assess recent dietary intake. Participants will have a venous blood sample (36ml) collected via venepuncture. Blood samples will be processed and stored in the Centre for Healthy Lungs Laboratory, Level 2, West Wing, HMRI. Medication use, occurrence of illness, and adverse events will be checked in conjunction with the completed trial diary.

Table 1. Schedule of Visits and Trial Procedures

	Visit:	Visit 0	Visit 1	PC1	Visit 2
Design	Time point	Screen	Day 0	Day 14	Day 28
	Visit Duration	0.5 hrs	1.0 hr	0.25 hrs	1.0 hr
	Randomisation		✓		
Questions	Eligibility assessment	✓			
	Informed consent		✓		
	Medical history	✓	✓		
	Medication use	✓	✓	✓	✓
	Smoking history		✓		
	Quality of Life (HRQOL-14)		✓		✓
	Food Frequency Questionnaire		✓		
	24 hr Food recall + frequency checklist		✓		✓
	Adverse events			✓	✓
Procedures	Blood collection and storage		✓		✓
	Height		✓		
	Weight		✓		✓
	Blood pressure		✓		✓
	Issue trial supplement		✓		
	Collect diary and trial supplement returns				✓

4.1.3 Outcome measures

Primary:

Ex-vivo production of IFN-γ by virus-infected PBMCs

Secondary:

Ex-vivo production of IL-6, IFN-α2, and TNFα by virus-infected PBMCs;

Peripheral immune cell subset numbers and percentages (leukocytes, granulocytes, eosinophils, neutrophils, T cells, CD4 T cells, CD8 T cells, Activated CD4 T cells, Activated CD8 T cells, Treg cells, γδ T cells, B cells, NK cells, BDCA-1 DCs, BDCA-3 DCs, pDCs);

Systemic inflammatory biomarkers (plasma IL-6, CRP, TNFα);

4.1.4 Measurement techniques

Blood collection: A total of 36ml of blood will be collected at each visit, in the HMRI building by the trained and qualified research assistant, following the venepuncture SOP (GCP009). The

volume of blood to be collected is less than 1% of total blood volume, and thus represents no hemodynamic risk to participants. Blood collection will be terminated if the participant experiences any adverse reactions or wishes to terminate the procedure. Blood samples will be processed and stored in the Centre for Healthy Lungs freezers on Level 2 West Wing, HMRI. Whole blood will be used for assessment of immune cell subsets by flow cytometry, plasma will be extracted and stored for biomarker analysis, then peripheral blood mononuclear cells (PBMCs) will be isolated to assess immune responses.

Immune cell profiling: Whole blood will be used for multi-parametric flow cytometry to determine the proportion and activation of immune cell subsets (**Table 2**). Whole blood will be incubated with fluorescently conjugated antibodies for specific cell surface antigens, then cells will be analysed using LSR Fortessa X-20 and FACSDiva software (BD Biosciences). Immune cell subsets will be determined based on differential antigen expression.

Table 2- Surface markers used to identify each cell subset

**Cell type**	**Surface markers**
Leukocytes	CD45+
Granulocytes	CD45+ SSC int/high
Eosinophils	CD45+ SSC int/high CD193high
Neutrophils	CD45+ SSC int/high CD193low
T cells	CD3+
CD4 T cells	αβ TCR+ CD4+
CD8 T cells	αβ TCR+ CD8+
Activated CD4 T cells	αβ TCR+ CD4+ CD25+ CD127 high/+
Activated CD8 T cells	αβ TCR+ CD8+ CD25+ CD127 high/+
Treg cells	αβ TCR+ CD4+ CD25+ CD127 low/-
γδ T cells	γδ TCR+ CD4+
B cells	CD3- CD19+
NK cells	CD3- CD56+ CD16+
BDCA-1 DCs	CD3- CD19- CD56- CD14- CD16- BDCA-1+
BDCA-3 DCs	CD3- CD19- CD56- CD14- CD16+ CD1c- CD303- BDCA-3high
pDCs	CD3- CD19- CD56- CD14- BDCA-2+

BDCA, blood dendritic cell antigen; DCs, dendritic cells; NK, natural killer; TCR, T-cell receptor; Treg, regulatory T cells; pDCs, plasmacytoid dendritic cells

Immune cell response: PBMC's will be isolated from whole blood using SepMate™ tubes. $4x10^6$ isolated PBMCs will be stored in buffer RLT for RNA extraction and gene expression analysis and additional analysis at the Kirby Institute, Sydney. Isolated PBMCs will be cultured and infected with human rhinovirus A16 (RV-16), and seasonal influenza (H1N1) for 48hrs, to examine anti-viral responses (IL-6, IFN-γ, IFN-α2, and TNFα) in cell culture supernatant by

bead-based multiplex assay (BD Bioscience). Supernatant will be stored in buffer RLT, and RNA and protein extracted from cultured cells will be stored for later analysis.

Gene expression analysis: RNA will be extracted from isolated PBMCs, cell culture supernatant and cultured cells (RNeasy Mini Kit, QIAGEN, Germany), then reverse transcribed to cDNA (High-Capacity cDNA Reverse Transcription Lot, Applied Biosystems, California, US). The expression of target mRNA will be measured using TaqMan® reagents (Applied Biosystems, California, US) with the ABI7500 Real Time PCR System. Protein concentration of genes of interest will be measured by ELISA.

Systemic inflammatory biomarkers: Whole blood will be centrifuged, plasma removed and stored at -80°C. Plasma concentrations of IL-6, CRP, TNFα will be analysed by high-sensitivity ELISA (Quantikine assays, R&D Systems).

Anthropometric measures: Anthropometric assessments (height and weight) will be performed at V1 and V2. Body mass index (BMI) will be calculated as follows: body weight (kg)/Height $(m)^2$.

Dietary intake: Participants will complete a food frequency questionnaire (FFQ) to assess usual dietary intake at baseline (DQES, Victorian Cancer Council). At each visit (V1 & V2) a 24-hour food recall with frequency checklist to reflect recent dietary intake will be collected by a research assistant trained in collecting this information. 24-hour food recalls and frequency checklists will be analysed for daily intake (serves/day) of dairy foods and cow's milk, with actual and usual intake (serves/day) recorded.

Health-related quality of life (HRQOL-14): Participants will complete the HRQOL-14 at each visit (V1 & V2) to measure and monitor changes in health-related quality of life, including physical and mental well-being and functioning (29).

4.1.5 Adherence

Adherence to the intervention will be monitored and assessed using the trial diary and pill countback of remaining trial supplement capsules at V2. Participants will be asked to complete the diary each day by recording consumption of the trial supplement.

4.2 Statistical Analysis Plan

Data will be analysed using STATA 15 (StataCorp, Texas, USA). Normality will be assessed using Shapiro-Wilk tests. Baseline comparisons will be performed using independent t tests or

Wilcoxon Rank Sum tests and Chi^2 tests as appropriate. For primary and secondary outcomes both intention to treat (ITT) (using all available data) and per protocol (PPA) analyses will be conducted. PPA will include participants with at least 90% compliance with the intervention, determined by pill count back. Linear mixed effects models (LMM) will be used to determine the group difference in change from baseline during the intervention, for continuous variables. LMM will be fit by restricted maximum likelihood, with group and time as fixed effects, time as a random effect and adjusted as necessary for any baseline differences. Changes within each treatment compared to baseline will be examined using paired Student's t test (normally distributed data) or Wilcoxon matched-pairs signed-rank tests (non-normally distributed data). Associations between continuous variables will be assessed using either Pearson's or Spearman's rank correlations. Significance will be accepted if $p<0.05$.

4.3 Sample size

Based on our previous studies, we can expect IFN-γ release from stimulated PBMCs to decrease by 0.8SD following supplementation. We would need n=31 subjects per group to detect this difference (80% power, $\alpha=0.025$). Allowing for 10% dropouts, we would require n=34 subjects per group, total n=102.

4.4 Randomisation

Eligible participants will be assigned a unique randomisation number using computer-generated codes, in variable size blocks, stratifying for age and gender. The randomisation service will be managed by an independent statistician at HMRI. During the intervention both the participants and the investigators will be blinded to the allocation.

5. Investigational product

5.1.1 Description

The Investigational product (IP) is bovine lactoferrin (200mg/d and 600mg/day) extracted from Australian cow's milk, containing 94% lactoferrin protein. Lactoferrin is a whey protein which normally accounts for 0.3% of the protein in cow's milk, at concentrations of 2-20 mg/100 ml. The IP will be supplied in 100mg/300mg gelatin capsules, along with identical placebo capsules containing only inert ingredients (microcrystalline cellulose, beta vulgaris root powder and maltodextrin).

5.1.2 Safety

The US Food and Drug Administration (FDA) has determined lactoferrin as Generally Recognised as Safe (GRAS). GRAS status is granted to a food ingredient by the FDA after extensive scientific evidence demonstrates that a product is safe for intended use. The FDA reported that no published evidence exists that bLf is a clinically relevant allergen.

In 1999, the Complementary Medicines Evaluation Committee of the Australian Therapeutic Goods Administration (TGA) were unable to find any reports of adverse events associated with bovine lactoferrin. The committee determined that bovine lactoferrin is of sufficiently low risk and is suitable for use in listable therapeutic goods. The Food Standards Australia New Zealand (FSANZ) has approved general level health claims for lactoferrin, including ‘contributes to healthy immune system function’; and ‘contributes to/supports optimal immune function’ and ‘provides enhanced protection from infections’.

5.1.3 Digestion, absorption and excretion

The digestion and metabolic fate of lactoferrin has been evaluated from studies of both human and bovine lactoferrin. Lactoferrin from both sources is handled similarly by the body. Lactoferrin is relatively poorly digested, with a substantial proportion of intact lactoferrin and its peptides persisting throughout the gastrointestinal tract, ultimately being excreted in the faeces. Lactoferrin and its peptides may also be partially absorbed from the gastrointestinal tract via specialized lactoferrin receptors and enter the blood. Lactoferrin is removed from the systemic circulation for distribution into organs and a proportion of the absorbed lactoferrin is ultimately excreted in the urine.

5.1.4 Manufacturer

The IP is manufactured and supplied by Noumi Limited. It is produced according to GMP in a Dairy Food Safety Victoria accredited facility, is Safe Quality Food registered, and Kosher and Halal certified. The trial supplement capsules are manufactured and packaged according to GMP in a TGA approved facility by BJP Laboratories Pty Ltd (Yatala, QLD, Australia), and labelled by Complementary Medicines Group (Warriewood, NSW, Australia).

5.1.5 IP Summary

**Approved Name**	**Bovine Lactoferrin**
**Trade Name (if any):**	PUREnFERRIN
**Manufacturer of IP**	Noumi Limited
**Approved therapeutic indication in Australia**	TGA Permitted Indications for Bovine Lactoferrin:<br>Antioxidant/Reduce free radicals formed in the body<br>Helps enhance/promote general health and wellbeing<br>Maintain/support general health and wellbeing<br>Maintain/support immune system health<br>Helps enhance/improve/promote immune system function<br>Helps stimulate a healthy immune system response
**Believed mode of action**	Bovine Lactoferrin is known to exert a variety of physiological functions, including antiviral, antimicrobial, antioxidant and immunomodulatory activities [2].
**Dosage regimen**	One daily dose of either 2x 100mg or 2 x 300mg in capsule form.
**Mode of excretion**	Lactoferrin is relatively poorly digested, with a substantial proportion of intact lactoferrin and its peptides persisting throughout the gastrointestinal tract, ultimately being excreted in the faeces. Lactoferrin and its peptides may also be partially absorbed from the gastrointestinal tract via lactoferrin receptors and enter the blood. Lactoferrin is removed from the systemic circulation for distribution into organs and a proportion of the absorbed lactoferrin is ultimately excreted in the urine.
**Known adverse events:**	Nil
**Known contra-indications or warnings:**	Contains cow's milk/milk products.
**Storage Conditions and Shelf Life**	Store in original sealed packaging in a pest free, cool, clean and dry environment < 25°C, at relative humidity of < 65%.<br>Use by 24 months from date of manufacture when unopened

6. Safety and Ethical Considerations

6.1 Safety

The conduct of the trial will be in accordance with the Code of Good Clinical Practice (GCP) and the NHMRC National Statement of Ethical Conduct in Research. The Investigators and trial personnel will meet regularly to monitor recruitment, progress, adverse events and data entry throughout the trial.

Participants will be closely monitored while undergoing testing. Only qualified staff who are fully trained in the operation of equipment, clinical procedures and response to emergencies will complete evaluations.

The side effects of having blood collected may include bleeding or bruising at the injection site and possible dizziness and/or fainting.

The trial supplement contains ingredients that are approved foods and considered safe and suitable for use in food in Australia. There are no known side effects.

With the participant's permission, abnormal findings from routine clinical tests will be forwarded to their GP for follow up.

Safety approval from the UoN safety committee will be obtained for all trial activities.

6.1.1 Reporting of Adverse Events

An Adverse Event (AE) is defined as any untoward medical occurrence in a participant during the assessments conducted at each clinic visit and during the trial intervention that may or may not be related to the trial protocol and/or investigational product. A Serious Adverse Event (SAE) is defined as any untoward serious medical occurrence at any dose that results in death, or is life-threatening or requires inpatient hospitalisation, or results in persistent or significant disability/incapacity, or is a congenital anomaly/birth defect, or is a medically important event or reaction. A Significant Safety Issue (SSI) is a safety issue that could negatively affect the safety of participants or impact the continued ethical acceptability or conduct of the trial. An unexpected and related SAE (URSAE) is an SAE that is both related to the intervention and not described in the protocol as an expected occurrence. A suspected unexpected serious adverse reaction (SUSAR) is an adverse reaction that is both serious and unexplained.

The trial coordinator will inform the principal investigator of all events as described above, who will then follow procedures for unblinding if necessary and notify the relevant bodies. Depending on the nature of event, it may be necessary for treatment to cease and/or for the participant to be withdrawn from the trial.

All SSIs will be documented on the SSI notification form and Case Report Form and will be reported within 72 hours to the Human Research Ethics Committee. All SUSARs will be documented on the SUSAR notification form and reported within 72 hours to the research governance office. Any complaints from participants in the research, or about the research, will

be documented in the Case Report Form and reported to the Human Research Ethics Committee.

6.1.2 Quality Control and Quality Assurance

Investigators and trial coordinators will be qualified and appropriately trained in the assessments of the trial. Data collection will be monitored by the principal investigator. A weekly update on the trials progress will be scheduled with the principal investigator and trial coordinator for monitoring purposes.

6.1.3 Discontinuation

Participants will be discontinued from the trial:

- Where indicated by the occurrence and nature of an adverse event
- The participant or the participant's general practitioner requests that the participant be withdrawn from the trial
- The participant refuses to comply with the requirements of the protocol
- If eligibility status changes during the trial, following diagnosis of new condition or changes to prescribed medications.

6.1.4 Disclosure, Publication and Confidentiality

Confidentiality of participants will be maintained. Patient identity will be limited to authorised staff working on this trial. Participants will be assigned a unique participant identification code. All data collected for the purposes of this trial will be kept a separate folder and participants will not be identified from these folders. Any reports and/or publication arising from this trial will only report average results and no identifiable individual data will be presented. During statistical data analysis the database will be stored in a password protected computer file. All data for the trial will be retained on file by the principal investigators at the University of Newcastle, in a locked data storage site for a period of 15 years.

6.1.5 Informed Consent, Ethical Review, and Regulatory Considerations

Participants will be given sufficient time after being given the trial patient information sheet to consider and discuss participation with friends and family. A contact number will be given to the potential participant, should they wish to discuss any aspect of the trial. Each participant will have the opportunity to have all their queries answered by the investigators prior to trial commencement and will not be coerced into signing the Consent Form. Following this, the

recruiting researcher will determine that the participant is fully informed of the trial in accordance with Good Clinical Practice guidelines. The right of the participant to refuse to participate in the trial without giving reasons will be respected. The participant will remain free to withdraw at any time from the trial, without giving reasons and without prejudicing his/her further care.

The informed consent process will be documented by the participant's dated signature on the Consent Form, which will be signed and dated by the investigator. The participant will receive a copy of the signed Consent Form and the Participant Information Statement. Consent will be taken by a member of the trial team who is trained in Good Clinical Practice.

The trial will not commence until full approval has been granted by the Hunter New England Human Research Ethics Committee and the trial has been registered with the ANZCTR. After obtaining full ethics approval, a Clinical Trials Notification (CTN) form will be lodged with the Department of Health and Ageing Therapeutic Goods Administration (TGA) for the use of the investigational product in this trial. This trial will be conducted in accordance with the ethical principles stated in the Declaration of Helsinki or the applicable guidelines of the Good Clinical Practice, which ever represents the greater protection of the individual.

7. References

1. Meyer, KC. Aging. Proc Am Thorac Soc. 2005;2(5):433-439.

2. Wakabayashi, Het al. Lactoferrin research, technology and applications. International Dairy Journal. 2006 2006/11/01/;16(11):1241-1251.
